# Widespread Arginine Phosphorylation in *Staphylococcus aureus*

**DOI:** 10.1016/j.mcpro.2022.100232

**Published:** 2022-04-12

**Authors:** Nadine Prust, Pieter C. van Breugel, Simone Lemeer

**Affiliations:** 1 Biomolecular Mass Spectrometry and Proteomics, Bijvoet Center for Biomolecular Research and Utrecht Institute for Pharmaceutical Sciences, Utrecht University, Utrecht, The Netherlands; 2 Netherlands Proteomics Center, Utrecht, The Netherlands

**Keywords:** agrinine phosphorylation, staphylococcus aureus, LC-MS/MS, Fe^3+^-IMAC, Stp1, AGC, automatic gain control, ETD, Electron transfer dissociation, FA, Formic acid, HCD, Higher energy collision–induced dissociation, HSD, honestly significant difference, IMAC, Immobilized metal ion affinity chromatography, PSM, peptide-spectrum match, RT, retention time, SDC, sodium deoxycholate, TCS, two-component system

## Abstract

Arginine phosphorylation was only recently discovered to play a significant and relevant role in the Gram-positive bacterium *Bacillus subtilis*. In addition, arginine phosphorylation was also detected in *Staphylococcus aureus*, suggesting a widespread role in bacteria. However, the large-scale analysis of protein phosphorylation, and especially those that involve a phosphoramidate bond, comes along with several challenges. The substoichiometric nature of protein phosphorylation requires proper enrichment strategies prior to LC-MS/MS analysis, and the acid instability of phosphoramidates was long thought to impede those enrichments. Furthermore, good spectral quality is required, which can be impeded by the presence of neutral losses of phosphoric acid upon higher energy collision–induced dissociation. Here we show that pArg is stable enough for commonly used Fe^3+^-IMAC enrichment followed by LC-MS/MS and that HCD is still the gold standard for the analysis of phosphopeptides. By profiling a serine/threonine kinase (Stk1) and phosphatase (Stp1) mutant from a methicillin-resistant *S. aureus* mutant library, we identified 1062 pArg sites and thus the most comprehensive arginine phosphoproteome to date. Using synthetic arginine phosphorylated peptides, we validated the presence and localization of arginine phosphorylation in *S. aureus*. Finally, we could show that the knockdown of Stp1 significantly increases the overall amount of arginine phosphorylation in *S. aureus*. However, our analysis also shows that Stp1 is not a direct protein-arginine phosphatase but only indirectly influences the arginine phosphoproteome.

## Highlights

• Extensive protein arginine phosphorylation in *Staphylococcus aureus*.
• pArg phosphorylation is stable under common phosphor enrichment conditions.
• Arginine phosphorylation is as widespread as threonine phosphorylation.
• Phosphatase Stp1 indirectly influences the pArg phosphoproteome in *S. aureus*.

## Graphical Abstract

**Figure:**
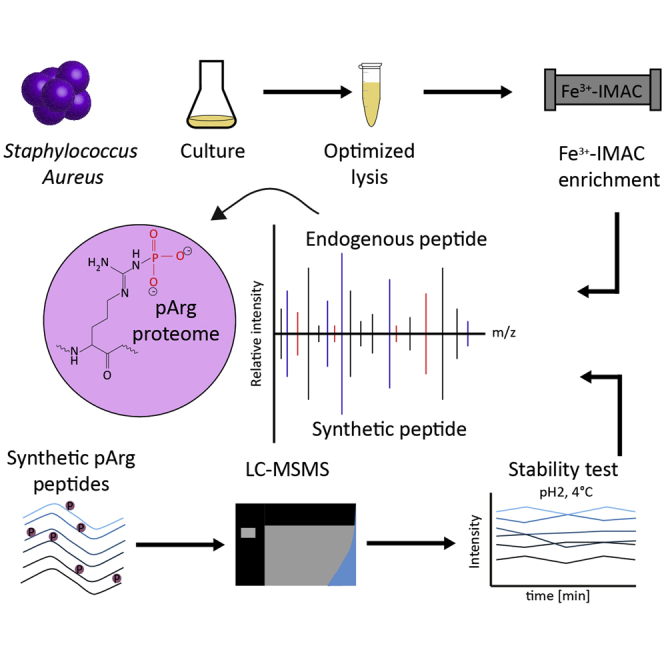

## In Brief

An optimized workflow for phosphopeptide enrichment revealed extensive protein arginine phosphorylation in *S. aureus*, which was shown to be stable under the enrichment conditions used. In *S. aureus* pArg phosphorylation is as widespread as pThr phosphorylation. The phosphatase Stp1 influences the arginine phosphoproteome in an indirect manner, increasing the number of proteins being pArg phosphorylated. It was shown that Stp1 is not a pArg phosphatase itself.

Protein phosphorylation is one of the most important reversible posttranslational modifications regulating a magnitude of cellular processes (1, 2). Hereto, a variety of protein kinases enable the transfer of the γ-phosphate of ATP to specific amino acid residues in the acceptor protein (3). The negative charge of the phosphoryl group can induce conformational changes that enable protein–protein interactions or changes in protein activity or subcellular localizations (4). Phosphatases can counteract this reaction by catalyzing the dephosphorylation of the proteins and thus allowing for a perfectly balanced on/off regulation of specific cellular processes. Eukaryotes mainly utilize phosphorylation on hydroxyl groups to form phosphomonoester (pSer, pThr, and pTyr) (5), whereas prokaryotes largely also exploit phosphorylation on amide groups to form phosphoramidates (pHis and pArg) (6, 7). Phosphoramidate bonds, contrary to phosphomonoester bonds, are rather unstable under acidic conditions (8, 9). Since most enrichment and fractionation strategies, prior to mass spectrometry–based analysis, are performed under strong acidic conditions those modifications remained long unidentified. This despite the notion that phosphoramidates, such as histidine phosphorylation, are known to fulfill important physiological roles, especially in bacteria and lower eukaryotes (6, 10). Recent studies on protein-histidine phosphorylation, however, showed that it is stable enough for commonly applied Fe^3+^–immobilized metal ion affinity chromatography (IMAC) enrichments (11, 12).

Protein-histidine phosphorylation is known to be involved in two-component system (TCS) signal transduction systems and plays a major role in prokaryotic signal transduction (6). TCS consists of a receptor histidine kinase sensing the outside environment and a corresponding response regulator, which mediates the intracellular response (6, 13). The autophosphorylated receptor histidine kinases transfers the phosphate group to an asparagine residue in the response regulator. Phosphorylation of the response regulator induces conformational changes that can trigger further downstream processes by, *e.g.*, binding to DNA (13). To date more than 16 histidine kinases are known for the Gram-positive bacterium *S. aureus* (13) and at least one of those TCS systems has been shown to cross talk with reversible phosphorylation *via* the Ser/Thr kinase Stk1 and the corresponding phosphatase Stp1 (14).

The other phosphoramidate, protein arginine phosphorylation, only received attention of the scientific community over the last decade after the identification of the first and yet only protein-arginine kinase, McsB, in the Gram-positive bacterium *Bacillus subtilis* (7). McsB shows homology to phosphagen kinases and was previously reported to act as tyrosine kinase (15, 16). The phosphatase YwlE was identified as tyrosine phosphatase and classified as low-molecular-weight protein tyrosine phosphatase based on its amino acid sequence (16, 17, 18). However, Elsholz *et al.* (19) could show that YwlE acts as protein-arginine phosphatase and thus counteracts the protein-arginine kinase activity of McsB.

Adaptations of common enrichments strategies to less acidic conditions (20, 21) or using a phosphatase trap mutant (22) enabled first insights in the arginine phosphoproteome of Gram-positive bacteria, resulting in the identification of around 200 pArg sites for *B. subtilis* (8, 19) and *S. aureus* (20, 21). Arginine phosphorylation was shown to play a functional role in the stress response, as well as in degradation pathways by marking proteins for degradation within the ClpC-ClpP proteasome, such as the transcriptional repressor CtsR (7, 19, 23, 24). Similar to histidine phosphorylation, reports of arginine phosphorylation in eukaryotes are scarce. A few studies reported the presences of pArg in rat liver (25) or mouse leukemia cells (26); however, a protein-arginine kinase has not been identified yet.

In addition to suitable enrichment strategies, the right fragmentation and automated spectrum interpretation are crucial for the identification and localization of arginine phosphorylated peptides. Higher energy collision–induced dissociation (HCD) fragmentation is known to generate extensive neutral loss fragments and therefore hampers the exact phosphosite localization by generating “unmodified” peptides. Electron transfer dissociation (ETD) in contrast, is preserving labile phosphosites and thus allows an unambiguous phosphosite localization (27, 28). ETD fragmentation has especially been shown to be beneficial for preservation of the unstable phosphoramidate bond of pArg during fragmentation (29).

Using an optimized sample preparation and subsequent Fe^3+^-IMAC enrichment we could show extensive protein-arginine phosphorylation in *S. aureus*, being as prevalent as threonine phosphorylation. Knockdown of the serine/threonine phosphatase Stp1 seemed to increase the amount of arginine phosphorylated proteins. Using synthetic peptides, we confidentially validated the presence of pArg phosphorylation and concomitantly analyzed different fragmentation methods for confident localization of arginine phosphorylation. Finally, using synthetic peptides and purified recombinant Stp1, we could convincingly show that Stp1 is not a direct arginine phosphatase but rather has a secondary effect on the arginine phosphoproteome.

## Experimental Procedures

### Bacterial Culture

*B. subtilis* 168 was grown overnight in 50 ml Luria broth (LB) at 37 °C with agitation in n = 3 biological replicates. Transposon mutants NE98 (disruption in unrelated surface protein encoding gene *sdrE*), NE217 (disruption in protein coding gene *pknB*), and NE1919 (disruption in gene SAUSA300_1112 encoding Stp1), all containing an erythromycin resistance marker, of the *S. aureus* USA300 JE2 strain were obtained from the Nebraska Transposon Mutant Library (30). Mutants were grown in n = 4 biological replicates, overnight in 25 ml Todd Hewitt broth supplemented with 5 μg/ml erythromycin at 37 °C with agitation as described (11, 31). Bacteria were harvested by centrifugation (15 min, 3200 rpm at 4 °C), and the supernatant was subsequently removed.

### Optimized Cell Lysis

Bacterial cell lysis was performed as described in Potel *et al.* (2018) (11) with optimization for Gram-positive bacteria. One volume bacteria pellet was resuspended in five volumes of lysis buffer (100 mM Tris-HCl pH 8.5, 7 M Urea, 5 mM tris(2-carboxyethyl)phosphine [TCEP], 30 mM 2-Chloroacetamide [CAA], 10 U/ml DNase I, 1 mM magnesium chloride [Sigma-Aldrich], 1% (v/v) benzonase [Merck Millipore], 1 mM sodium orthovanadate, phosphoSTOP phosphatases inhibitors [Roche], and complete mini EDTA-free protease inhibitors). The lysis was performed by bead beating for 17.5 min (1.5 min on, 2 min off) at 2850 rpm (Disruptor Genie, Scientific Industries) in case of *B. subtilis* and 3200 rpm (Mini-Beadbeater-24, Bio Spec Products Inc) for *S. aureus*. Subsequently, the beads were pelleted by centrifugation (2 min at 3000 rpm), and 1% (v/v) Triton X-100 in case of *B. subtilis* and 1% (v/v) Triton X-100 plus 1% (v/v) sodium deoxycholate (SDC, final concentration) in case of *S. aureus* was added to the bacterial lysate. Complete lysis was reached by sonication for 45 min (20 s ON, 40 s off) using a Bioruptor Plus. Cell debris was removed by ultracentrifugation (45,000 rpm for 1 h at 4 °C). Protein concentration of the supernatant was determined *via* a bicinchonic acid assay. To decrease the SDC concentration to <0.4%, the supernatant was diluted 2.5 times with dilution buffer (100 mM Tris-HCl pH 8.5, 7 M Urea, 5 mM TCEP, 30 mM CAA, 1 mM magnesium chloride [Sigma-Aldrich], 1 mM sodium orthovanadate, phosphoSTOP phosphatases inhibitors [Roche], and complete mini EDTA-free protease inhibitors). Benzonase, 1% (v/v), was added to the supernatant mixture and incubated for 2 h at room temperature. Subsequently, methanol/chloroform precipitation was performed as described (11). The precipitate was then resuspended in digestion buffer (100 mM Tris-HCL pH 8.5, 30 M CAA, 1% (v/v) SDC [Sigma-Aldrich], and 5 mM TCEP). Protein digestion was performed overnight at room temperature using a mix of trypsin and Lys-C in a ratio of 1:25 and 1:100 (w/w), respectively. Protein digests were acidified to pH 3.5 using 10% formic acid (Sigma-Aldrich), and precipitated SDC was removed by centrifugation (1400 rpm, 5 min). The supernatant was loaded onto C18 Sep-Pak (3 cc) resin columns (Waters) for desalting. The loaded samples were washed twice with 0.1% (v/v) formic acid, and bound peptides were eluted with 600 μl 40% acetonitrile and 0.06% formic acid. Eluted peptides were split into 2-mg fractions, and samples for full proteome analysis were frozen in liquid nitrogen and freeze dried.

### Phosphopeptide Enrichment

Fe^3+^-IMAC enrichments were performed as described (11). In short, 2 mg lyophilized peptides were resuspended in loading buffer A (30% acetonitrile and 0.07% TFA) and, if necessary, the pH was adjusted to 2.3 using 10% TFA. The samples were loaded onto the Fe^3+^-IMAC column (Propac IMAC-10 4 × 5 mm column, Thermo Fischer Scientific). Bound phosphopeptides were eluted with elution buffer B (0.3% NH_4_OH). The respective gradient is described in supplemental Table S1. The UV-abs signal at a wavelength of 280 nm was recoded at the outlet of the column, and eluting phosphopeptides were collected manually. Subsequently, phosphopeptides were frozen in liquid nitrogen and freeze dried.

### Liquid Chromatography/Tandem Mass Spectrometry

Nanoflow liquid chromatography/ tandem mass spectrometry (LC-MS/MS) analysis was performed using an Agilent 1290 (Agilent technologies) coupled to an Orbitrap Q-Exactive HF-X (Thermo Fisher Scientific). Lyophilized phosphopeptides or full proteome samples were resuspended in 20 mM citric acid (Sigma-Aldrich), 1% (v/v) formic acid, or 2% (v/v) formic acid, respectively. Resuspended phosphopeptide, corresponding to 1.6 mg or 200 ng full proteome samples were injected, trapped, and washed on a trap-column (100 μm i.d. × 2 cm, packed with 3 μm C18 resin, Reprosil PUR AQ, Dr. Maisch, packed in-house) for 5 min at a flow rate of 5 μl/min with 100% buffer A (0.1 formic acid [FA], in HPLC grade water). Peptides were subsequently transferred onto an analytical column (75 μm × 60 cm Poroshell 120 EC-C18, 2.7 μm, Agilent Technology, packed in-house) and separated at room temperature at a flow rate of 300 nl/min using an 85-min linear gradient from 8% to 32% buffer B (0.1% FA, 80% ACN) or a 115-min linear gradient from 13% to 44% buffer B. Electrospray ionization was performed using 1.9 kV spray voltage and a capillary temperature of 320 °C. The mass spectrometer was operated in data-dependent acquisition mode: full scan mass spectrometry (MS) spectra (*m/z* 375–1600) were acquired in the Orbitrap at 60,000 resolution for a maximum injection time of 20 ms with an automatic gain control (AGC) target value of 3e6 charges. Up to 12 precursors for phosphoproteome samples and up to 15 precursors for full proteome samples were selected for subsequent fragmentation. High-resolution HCD MS2 spectra were generated using a normalized collision energy of 27%. The intensity threshold to trigger MS2 spectra was set to 2e5, and the dynamic exclusion to 12 or 16, respectively. MS2 scans were acquired in the Orbitrap mass analyzer at a resolution of 30,000 (isolation window of 1.4 Th) with an AGC target value of 1e5 charges and a maximum ion injection time of 50 ms. Precursor ions with unassigned charge state as well as charge state of 1+ or superior/equal to 6+ were excluded from fragmentation.

### *E. coli* Samples

*E. coli* samples published by Potel *et al.* (11) were reanalyzed for pArg. Detailed sample preparation can be found in the respective publication. In short, *E. coli* strain W3110 was used and grown in M9 minimal medium, consisting of M9 salts (6 g/l Na2HPO4, 3 g/l KH2PO4, 0.5 g/l NaCl, 1 g/l NH4Cl) supplemented with additional 0.5% (w/v) glucose, 1 mM MgSO4, 0.1 mM CaCl2, with vigorous shaking at 37 °C. Cells were collected by centrifugation at stationary phase (*A*_600_ = 1.2) and washed three times with ice-cold PBS. Samples were lysed as described above under optimized sample preparation, however, without the addition of SDC and additional bead beating.

### Synthetic Peptides

#### Synthetic Arginine Peptide Analysis

Seventeen synthetic peptides phosphorylated on arginine were ordered from Pepscan (supplemental Table S2). Counterparts phosphorylated on Ser, Thr, or Tyr were ordered from JPT Peptide Technologies GmbH, Berlin, Germany (supplemental Table S2). Peptides were reconstituted in 0.1 M Ammonium bicarbonate, 20% acetonitrile and diluted to 400 fmol/μl. A peptide mix containing all 17 pArg-peptides was prepared with adjusted concentrations for best peptide identification. All 45 pSTY peptides were mixed in a 1:1 ratio of 400 fmol per peptide.

#### Stability Test of Synthetic pArg-Peptides

Synthetic peptides (n = 4) were constituted in 52.5 μl 20 mM citric acid (Sigma-Aldrich), 2% (v/v) FA and 250 ng *E. coli* digest was spiked in. The peptide mix was spun down for 3 min at 20,817*g*, 4 °C and loaded on the plate 0, 15, 30, 60, or 120 min prior to LC-MS/MS analysis. Nanoflow LC-MS/MS analysis was performed using an Agilent 1290 (Agilent Technologies) coupled to an Orbitrap Fusion Lumos (Thermo Fisher Scientific). Resuspended phosphopeptides, corresponding to 16% of the peptide mix were injected, trapped, and washed on a trap-column (100 μm i.d. × 2 cm, packed with 3 μm C18 resin, Reprosil PUR AQ, Dr. Maisch, packed in-house) for 5 min at a flow rate of 5 μl/min with 100% buffer A (0.1 FA, in HPLC grade water). Peptides were subsequently transferred onto an analytical column (75 μm × 60 cm Poroshell 120 EC-C18, 2.7 μm, Agilent Technology, packed in-house) and separated at room temperature at a flow rate of 300 nl/min using a 40-min linear gradient from 10% to 40% buffer B (0.1% FA, 80% ACN). Electrospray ionization was performed using 1.9 kV spray voltage and a capillary temperature of 320 °C. The mass spectrometer was operated in data-dependent acquisition mode: full scan MS spectra (*m/z* 375–1500) were acquired in the Orbitrap at 60,000 resolution for a maximum injection time of 50 ms with an AGC target value of 4e5 charges. High-resolution HCD MS2 spectra were generated using a normalized collision energy of 35%. The intensity threshold to trigger MS2 spectra was set to 5e5, and the dynamic exclusion 2. MS2 scans were acquired in the Orbitrap mass analyzer at a resolution of 30,000 (isolation window of 1.6 Th) with an AGC target value of 1e5 charges and a maximum ion injection time of 100 ms. Precursor ions with unassigned charge state as well as charge state of 1+ or superior/equal to 6+ were excluded from fragmentation.

Retention time (RT) analysis was performed using an Ultimate 3000 (Thermo Fisher Scientific) coupled to an Orbitrap Exploris 480 (Thermo Fisher Scientific). Resuspended phosphopeptides, corresponding to 16% of the pArg-peptide mix or 8% pSTY peptide mix both containing 4 ng/μl *E. coli* digest were injected, trapped, and washed on a trap-column (μ-Precolumn, 300 μm i.d. × 5 mm C18 PepMap100, 5 μm, 100 Å [Thermo Scientific, P/N 160454]) for 5 min at a flow rate of 5 μl/min with 92% buffer A (0.1 FA, in HPLC grade water). Peptides were subsequently transferred onto an analytical column (75 μm × 50 cm Poroshell 120 EC-C18, 2.7 μm, Agilent Technology, packed in-house) and separated at 40 °C at a flow rate of 0.3 μl/min using a 40-min linear gradient from 9% to 36% buffer B (0.1% FA, 80% ACN). Electrospray ionization was performed using 1.9 kV spray voltage and a capillary temperature of 275 °C. The mass spectrometer was operated in data-dependent acquisition mode: full scan MS spectra (*m/z* 375–1600) were acquired in the Orbitrap at 60,000 resolution for a maximum injection time set to auto-mode with a standard AGC target. High-resolution HCD MS2 spectra were generated using a normalized collision energy of 28%. The intensity threshold to trigger MS2 spectra was set to 5e4, and the dynamic exclusion 2. MS2 scans were acquired in the Orbitrap mass analyzer at a resolution of 30,000 (isolation window of 1.4 Th) with a normalized AGC target of 200% and an automatic maximum injection time. Precursor ions with unassigned charge state as well as charge state of 1+ or superior/equal to 6+ were excluded from fragmentation.

#### ETD, EThcD, and HCD Fragmentation

Nanoflow LC-MS/MS analysis was performed using an Agilent 1290 (Agilent technologies) coupled to an Orbitrap Fusion (Thermo Fisher Scientific). Resuspended phosphopeptide, corresponding to 80% of the peptide mix were injected, trapped, and washed on a trap-column (100 μm i.d. × 2 cm, packed with 3 μm C18 resin, Reprosil PUR AQ, Dr. Maisch, packed in-house) for 5 min at a flow rate of 5 μl/min with 100% buffer A (0.1 FA, in HPLC grade water). Peptides were subsequently transferred onto an analytical column (75 μm × 50 cm Poroshell 120 EC-C18, 2.7 μm, Agilent Technology, packed in-house) and separated at room temperature at a flow rate of 300 nl/min using a 40-min linear gradient from 10% to 40% buffer B (0.1% FA, 80% ACN). Electrospray ionization was performed using 2 kV spray voltage and a capillary temperature of 275 °C. The mass spectrometer was operated in data-dependent acquisition mode: full scan MS spectra (*m/z* 375–1500) were acquired in the Orbitrap at 60,000 resolution for a maximum injection time of 50 ms with an AGC target value of 4e5 charges. High-resolution EThcD spectra were generated using a supplemental activation collision energy of 35%, and high-resolution HCD spectra were generated using a normalized collision energy of 35%. For all three fragmentation methods, the intensity threshold to trigger MS2 spectra was set to 5e5, and the dynamic exclusion 2. MS2 scans were acquired in the Orbitrap mass analyzer at a resolution of 30,000 (isolation window of 1.6 Th) with an AGC target value of 1e5 charges and a maximum ion injection time of 100 ms. Precursor ions with unassigned charge state as well as charge state of 1+ or superior/equal to 6+ were excluded from fragmentation.

### Plasmid Construction

To generate pET28a(+)-EGFP, the eGFP gene was excised out from pEGFP-N3 (Clontech/TaKaRa) by restriction digestion and cloned into pET28a(+) (Merck-Novagen, Cat# 69864) using BamHI and NotI (Thermo Fisher Scientific). To generate expression vectors pET28a(+) Stp1 and pET28a(+) PknB/Stk1, respective genes were amplified by PCR using chromosomal DNA form *S. aureus* as template and indicated primers ordered at Merck-Sigma (supplemental Table S3). PCR-generated DNA fragments were cloned into pET28a(+) by restriction digestion using NdeI and BamHI. All constructs were sequence verified by Sanger sequencing.

### Protein Overexpression and Purification

For the preculture 20 ml YT 2× medium supplemented with 50 μg/ml kanamycin and 25 μg/ml chloramphenicol were inoculated with BL21(DE3)pLysS + pET28 (+), BL21(DE3)pLysS + pET28 (+)eGFP, or BL21(DE3)pLysS + pET28 (+)Stp1 and incubated overnight at 37 °C, 190 rpm. Precultures were pelleted at 4816*g* for 15 min, the pellets were suspended in 20 ml fresh medium, and *A*_600_ was measured. The 20 ml precultures were added to 800 ml YT 2× medium supplemented with 50 μg/ml kanamycin and 25 μg/ml chloramphenicol and incubated at 37 °C, 190 rpm until an *A*_600_ of 0.6 was reached. Protein expression was induced by adding a final concentration of 1 mM IPTG. Proteins were expressed for approximately 16 h; overexpression was confirmed by SDS-PAGE and subsequent staining with Imperial Protein Stain (Thermo Fischer Scientific).

For the protein purification, pellets of a 400 ml culture were suspended in 5 ml lysis buffer per g wet cell paste (50 mM Sodium dihydrogen phosphate (NaH2PO4), 0.5 M sodium chloride (NaCl), 1% (v/v) Triton X-100, 0.1% (v/v) benzonase [Merck Millipore], 10 μ/ml DNase 1, 2 mM magnesium chloride [Sigma-Aldrich], and completeMini EDTA-free protease inhibitors [Roche]). Samples were incubated for 1 h on ice before sonication was performed using the Dr. Hielscher, UP100H with Micro tip MS3 (five cycles of 1 min on: cycle 0.5, amplitude 90% and 1.5 min off). Insoluble parts and cell debris were pelted for 30 min at 4694*g* at 4 °C, and the soluble fraction was taken for purification using Ni-NTA Agarose beads (Qiagen). A volume of 200 μl Ni-NTA beads was loaded on Bio-Rad Poly-Prep Columns 10 ml and first equilibrated with 600 μl distilled water and subsequently with 1× binding buffer (0.5 M NaCl, 20 mM Tris-HCl pH 8, 5 mM imidazole). The soluble fraction was supplemented with a final concentration of 5 mM imidazole, loaded on the column, and incubated for 1 h a 4 °C over-head shaking. The beads were washed twice with 1 ml 1× binding buffer and 4× with 500 μl 1× wash buffer (0.5 M NaCl, 30 mM imidazole, and 20 mM Tris-HCL pH 8). Bound proteins were eluted five times with 500 μl 1× elution buffer (0.5 M imidazole, 0.5 M NaCl, and 20 mM Tris-HCl pH 8). To confirm the purification an SDS-PAGE with subsequent staining using Imperial Protein Stain (Thermo Fischer Scientific) was performed. To stabilize the purified protein 50% glycerol was added to the Stp1 eluates.

### Dephosphorylation Assay

Synthetic pArg, 24.8 pmol, or 22.4 pmol pSTY peptides (n = 4) were reconstituted in 100 μl reaction buffer (50 mM Tris-HCl pH 8, 100 mM NaCl, 2 mM Manganese(II) chloride (MnCl2), 1 mM dithiothreitol). Peptide solutions were incubated for 1 h at 20 °C either with 2 μg Stp1, 10 units Shrimp alkaline phosphatase (New England BioLabs Inc) or without the addition of any phosphatase. Subsequently the samples were lyophilized. For LC-MS/MS analysis synthetic pArg-peptides were reconstituted as described for the stability analysis.

### Data Analysis

Raw files were processed using MaxQuant software (version 1.6.3.4 and 1.6.17.0 for HCD data and version 1.5.3.30 for ETD, EThcD, and HCD data), and the Andromeda search engine was used to search against *S. aureus* USA300 (Uniprot, June 2018, 5954 entries), *E. coli* (Uniprot, March 2016, 4434 entries), or *B. subtilis* (Uniprot/TrEMBL, December 2017, 4247 entries) or a data base containing all 17 synthetic pArg-peptides with the following parameters for phosphoproteome analysis: trypsin digestion with a maximum of three missed cleavages, carbamidomethylation of cysteines (57.02 Da) as a fixed modification, methionine oxidation (15.99 Da), N-acetylation of proteins N termini (42.01 Da), and phosphorylation on serine, threonine, tyrosine, histidine, and arginine residues (79.96 Da) in case of pArg searches as variable modifications. Mass tolerance was set to 4.5 ppm at the MS1 level and 20 ppm at the MS2 level. The false discovery rate was set to 1% for peptide-spectrum matches (PSMs) and protein identification using a target-decoy approach, a score cutoff of 40 was used in the case of modified peptides, and the minimum peptide length was set to seven residues. The match between run feature was enabled with a matching time window of 0.7 min and an alignment time window of 20 min for the endogenous phosphoproteome. The MaxQuant generated tables “evidence.txt” and “phospho (HSTY)Sites.txt” were used to calculate the number of unique phosphopeptides and phosphosites identified, respectively, and known contaminants were filtered out. For full proteome analysis the following deviations were applied: trypsin digestion with a maximum of two missed cleavages, carbamidomethylation of cysteines (57.02 Da) as a fixed modification, methionine oxidation (15.99 Da), and N-acetylation of protein N termini (42.01 Da) as variable modifications. Relative label-free quantification was performed using the MaxLFQ algorithm with the minimum ratio count set to 2.

### Statistical Data Analysis of Endogenous Peptides

All used scripts are published (https://github.com/hecklab/Protein-arginine-phosphorylation) and can be downloaded. For more detailed information on the endogenous data we refer you to our previous study (31). The MaxQuant generated “phospho (RHSTY)Sites.txt” and “proteinGroups.txt” files, respectively, were used for subsequent statistical data analysis in R studio (R version 3.6.0). Four biological replicates per mutant were analyzed. The data were filtered for “Reversed” and “Potential contaminant.” In case of the phosphoproteome, an Andromeda localization score greater than 0.75 was required. Intensities for the phosphoproteome data, or LFQ intensities in case of the full proteome analysis, were log2 transformed. For each phosphosite or protein, the median calculated per sample was subtracted to compensate for systematic measurement effects: only proteins with at least three valid values in one condition and two valid values in at least one other condition. Data were checked for normal distribution before one-way ANOVA on each phosphosite or protein was done, after which the *p*-values were adjusted with the Benjamini–Hochberg procedure. The post hoc Tukey Honestly Significant Difference (HSD) method was used to identify changing p-sites between the individual groups. A Tukey HSD *p*-value cutoff of 0.05 and a fold change cutoff of the mean ± one standard deviation of the data were used to select for significantly changing phosphosites or proteins between two groups.

### Experiment Design and Statistical Rationale

Each sample was grown in n = 4 biological replicates, enriched, and injected separately into the LC-MS/MS system. Synthetic peptides were run in n = 4 technical replicates. Each raw file was separately processed using the MaxQuant software. This analysis was sufficient to saturate the number of phosphosites detected.

### GO-Term Analysis

All identified pArg proteins were used to identify overrepresented GO terms and protein classes using PANTHER (32). For the Fischer exact test, the whole *S. aureus* genome (2889 entries) was used as a background. Only genes having a gene name and not only a gene Locus ID were considered (74 genes). As comparison all pST (303 genes) proteins were also used for an enrichment. A false discovery rate cutoff of 0.05% was used.

### Analysis of Synthetic pArg-Peptides

To analyze the stability of synthetic arginine phosphorylation, the MaxQuant generated evidence.txt and “msms.txt” files were used within the R studio (R version 3.6.0). Four technical replicates per time point were analyzed. The data were filtered for “Reversed,” “Potential contaminant,” “MULTI-MSMS” spectra and the intensity was log2 transformed. Identifications in the evidence.txt and msms.txt files were linked *via* the “Best.MS.MS” identifier.

#### Stability Test

The intensities of unique peptides were summed, and the average per time point was calculated. Unique peptides are defined based on the Modified sequence. The ratio for pArg to unmodified peptides was determined as the difference between the respective log2 intensity averages per peptides.

#### Retention Time Analysis

The average RT per unique peptide was calculated taking different charge states into account. The RTs of pArg-peptides were plotted as function of the RT of unmodified peptides. Respectively, peptides identified only as arginine phosphorylated or unmodified were not considered for the analysis. Further pArg and pSTY peptide mixes were spiked in with 1 μl of 1× iRT peptides and the RT of pArg-peptides were plotted as function of the RT of synthetic pSTY peptides. iRT peptides were used to compare the RT between different liquid chromatography runs.

#### Fragmentation Analysis

The sequence coverage for the three different fragmentation methods was calculated based on all ions contributing to the sequence coverage. For HCD y- and b-ions as well as the respective neutral-loss y∗ ions were considered. For ETD c- and z-radical ions as well as the less common c-radical and z-ions were considered. For EThcD, respectively, all of the above-mentioned ions were considered. In case one fragment ions was identified based on different types, *e.g.*, y2 and y∗2; this was counted as one ion to calculate the percentage based on the theoretically observed number of ions. The average sequence coverage, Andromeda score, and localization probability were calculated for each fragmentation method as well as for each unique peptide.

#### Neutral-Loss and Immonium-Ion Analysis

Raw files from the fragmentation analysis and endogenous samples were converted into .mgf with Proteome Discoverer (Vers. 2.3.0.523). Subsequently mgf-files were analyzed using an in-house made script searching PSMs matching the different phosphorylated amino acids using the MaxQuant generated evidence.txt and “msmsScans.txt” file for neutral-loss ions (HPO_3_ = 79.966331 Da, H_3_PO_4_ = 97.976896 Da, and H_5_PO_5_ = 115.98746 Da) and potential immonium ions.

To analyze the occurrence of potential immonium ion the extracted MS2 spectra matching the respective phosphorylated amino acids were searched with an in-house made script for potential pArg immonium ions (237.0747 *m/z* or 209.0798 *m/z*) and pHis immonium ion (190.0376 *m/z*).

#### Spectra Comparison

To compare MS2 spectra from the synthetic pArg-peptides with the endogenous identified counterparts, the MaxQuant generated evidence.txt files were loaded in an in-house developed software FragmentLab (https://scheltemalab.com/software) (v2.4.1.0). The synthetic peptides were used to create a peptide library that was used to validate the endogenous identified pArg-peptides. FragmentLab calculates a quality score for the spectra comparison. Spectra mass lists were exported to plot the spectra. All comparisons for identified endogenous and synthetic peptides were extracted.

## Results and Discussion

### Stp1 Transposon Mutant Shows Extensive pArg Phosphorylation

Protein-arginine phosphorylation in Gram-positive bacteria gained attention since its physiological relevance for the bacterial stress response was shown in *B. subtilis* (8, 19). In this system, arginine phosphorylation is regulated by the only known protein-arginine kinase McsB and its respective protein-arginine phosphatase YwlE (7, 19). By analyzing a *S. aureus* COL ΔptpB mutant, Junker *et al.* (21) showed that the previous annotated tyrosine phosphatase PtpB acts as protein-arginine phosphatase and therefore counterbalances the staphylococcal protein-arginine kinase McsB (21). They exclusively identified 207 pArg sites in the ΔptpB mutant, which represented the largest staphylococcal arginine phosphoproteome (21). Spurred by this observation we searched for arginine phosphorylation in three different *S. aureus* mutant strains (31) and found that arginine phosphorylation is even more abundant than previously shown in *S. aureus*.

**Fig. 1:**
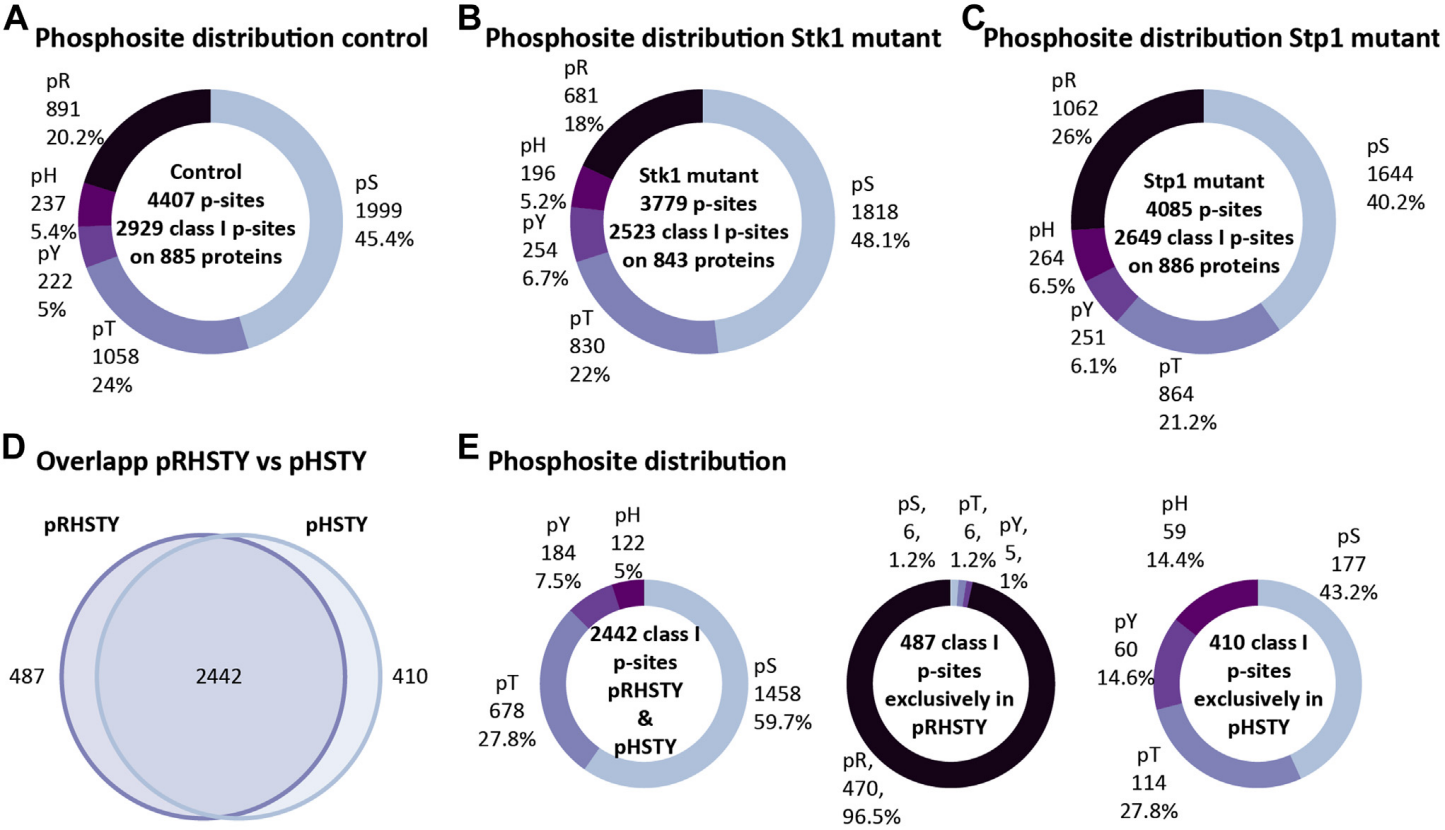
**Arginine phosphorylation distribution in the control, Stk1 mutant, as well Stp1 mutant of *S. aureus* and comparison of identified class I p-sites after MaxQuant search for pRHSTY and pHSTY for the control (n = 4).***A*–*C*, number of identified phosphosites, class I phosphosites (Andromeda localization probability >0.75), and phosphoproteins, as well as the distribution of serine, threonine, tyrosine, histidine, and arginine phosphorylation in the different mutant strains. *D*, overlap of identified class I p-sites after either pRHSTY or pHSTY search. *E*, phosphorylation site distribution for the 2442 p-sites identified in both searches as well as for the 487 and 410 p-sites identified exclusively after pRHSTY or pHSTY search, respectively.

Surprising, we found 891 pArg sites (470 class I, see supplemental Table S4) in the NE98 mutant (lacking the membrane protein SdrE), making arginine phosphorylation almost as abundant as threonine phosphorylation (45.4% pS, 24% pT, 5% pY, 5.4% pH, and 20.2% pR). Even more striking, we identified 1062 pArg sites (651 class I, see supplemental Table S4) in the Stp1 mutant (NE1919). With 26% of the phosphorylation sites localized on arginine, this is the second most abundant phosphorylation type after protein serine phosphorylation in this mutant (40.2% pS, 21.2% pT, 6.1% pY, 6.5% pH, and 26% pR) (Fig. 1*C*). In the Stk1 mutant 681 sites (350 class I) were identified (48.1% pS, 22% pT, 6.7% pY, 5.2% pH, and 18% pR). This makes the percentage of pArg sites in the Stp1 mutant 6% to 8% higher compared with the other mutants (Fig. 1, *A*–*C*). In addition, we also compared the intensity distribution of Ser, Thr, Try, His, and Arg phosphosites showing that pArg-sites do not have a higher intensity compared with the other phosphosites but are simply more widespread (supplemental Fig. S1).

Encouraged by the number of identified pArg-sites we also looked for arginine phosphorylation in other Gram-positive (*B. subtilis*) as well as Gram-negative (*E. coli*) bacteria. Even though *E. coli* possesses an arginine kinase homologous to McsB (see supplemental Fig. S2), here, we could identify less than 3% class I pArg-sites for *E. coli* (supplemental Fig. S2 and supplemental Table S4). For *B. subtilis* we could not identify any class I pArg-sites. This is in line with previous work where arginine phosphorylation could only be detected in the arginine phosphatase mutant (ΔYwlE) in *B. subtilis* (8). These results show that arginine phosphorylation seems to play a prominent role in *S. aureus* and that, unexpectedly, Stp1 appears to play a functional role in regulating arginine phosphorylation in *S. aureus*. This was further underlined by an increased number of exclusively arginine phosphorylated proteins in the Stp1 mutants (supplemental Fig. S3) compared with the control and the Stk1 mutant. Based on the data we obtained, we identified a small part of the proteome that is uniquely phosphorylated on an arginine residue (supplemental Fig. S3).

To avoid overinterpretation of these results we checked for pHSTY phosphorylated counterpart peptides of the identified pArg-peptides. For 386 of the 1294 identified pArg-peptides in at least one of the three mutants, we could identify a pHSTY counterpart. The Andromeda localization probability for those 386 pArg-peptides was lower than for their pHSTY counterparts (supplemental Fig. S4*A*). However, for peptides exclusively identified as pArg the Andromeda localization probability was even slightly better than for pHSTY peptides. The average Andromeda score distribution was similar between all phosphopeptides (supplemental Fig. S4*B*).

To further disprove that the identified pArg-sites are not the result of mislocalization we compared the class I sites identified for pRHSTY and pHSTY searches in the control (SdrE) mutant (31) and found that 83% of the identified sites overlapped. A total of 487 class I phosphosites were exclusively identified in the dataset searched for pRHSTY of which 96.5% were localized on Arg stressing that the larger search space almost exclusively gives rise to pArg sites (Fig. 1, *D* and *E*). Of these newly identified sites, 177 were reported to be phosphorylated on a different amino acid in the HSTY search, based on the identified peptides sequence (supplemental Fig. S5, *A* and *C*). We also identified a small number of class I sites that resulted in a different localization in the two searches, based on the same spectrum (supplemental Fig. S5*B*). Even though this shows a certain degree of mislocalization either toward pHis or pSTY, we see that the vast majority of pArg sites seem to be in fact newly (and additionally) identified phosphosites.

Phosphorylation on arginine presumably increases the number of missed cleavages after tryptic digestion. Trypsin, the most commonly used protease for shot-gun proteomics, forms a deep, narrow, negatively charged binding pocket that enables ionic interactions with long basic amino acids and thus cleaves specifically after lysine and arginine (33). The addition of a negatively charged phosphate group is therefore thought to impair the tryptic cleavage, and studies on arginine phosphorylation increased the number of allowed missed cleavages for database searches (8, 21, 34). Here, we show that indeed the majority of pArg-peptides (90%) showed at least one missed cleavage, whereas 70% of pHSTY peptides were identified without missed cleavage. For class I pArg-sites the percentage of nonmissed cleavage decreased even further and 97% of the identified peptides showed at least one missed cleavage (supplemental Fig. S6*A*). In addition, we also analyzed where the phosphorylation is located within the pArg-peptides. A comparison of all pArg sites and class I pArg sites showed that only a minority of class I pArg sites is located at the C terminus and that the majority is 1 to 5 amino acids away from the C terminus (supplemental Fig. S6*B*). These findings indeed confirm that phosphorylation of arginine impairs tryptic digestions, probably due to electrostatic repulsion and/or steric hindrance and in addition provides compelling evidence that identified (class I) pArg sites are not a product of mislocalization.

Arginine phosphorylation forms an acid-labile phosphoramidate bond, similar to histidine phosphorylation that was long thought to impede the enrichment and analysis of this posttranslational modification. In addition, and again similar to what is known for histidine phosphorylation, neutral loss of the phosphate group under fragmentation conditions might complicate correct site localization. Mislocalization of histidine phosphorylation is a major issue and can in some cases lead to extensive overestimation of the amount of phosphorylation. To exclude mislocalization, we carefully evaluated the behavior of arginine phosphorylated peptides under enrichment and fragmentation conditions, in order to provide confidence in proper site localization.

### Arginine Phosphorylation Is Stable Under Acidic Conditions

**Fig. 2:**
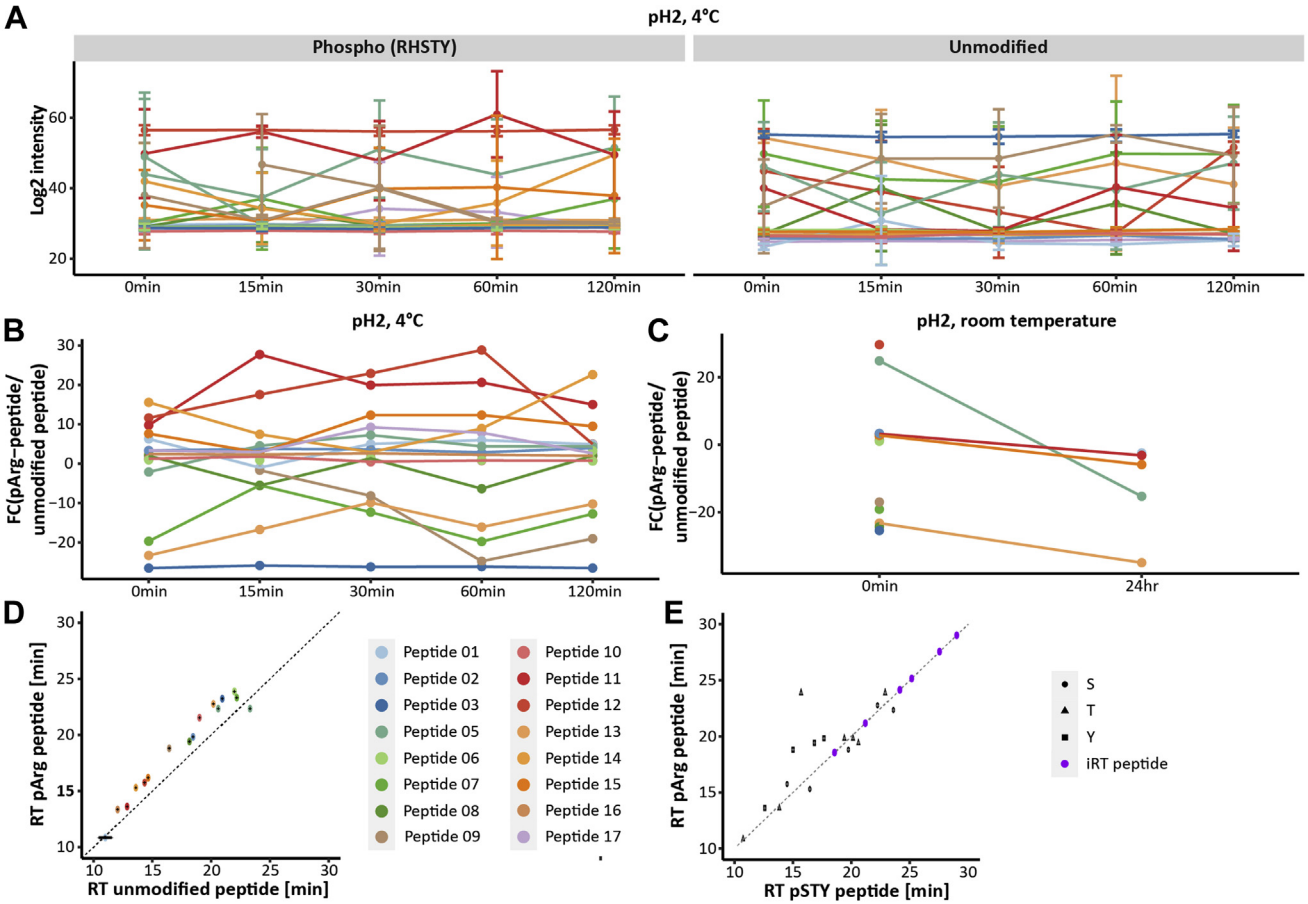
**Stability of synthetic arginine phosphorylate peptides.***A*, Log2 intensity of the 16 identified synthetic pArg-peptides over a time course of 120 min under acidic conditions (pH 2) at 4 °C and their respective unmodified counterpart. *B*, fold change of the respective pArg and unmodified peptides over the time course of 120 min at 4 °C. The ratio of pArg to unmodified peptide was also determined after incubating the peptides under acidic conditions (pH 2) at room temperature for 24 h. *C* and *D*, retention of pArg-peptides in relation to retention time of unmodified peptides. The *dashed line* shows the ideal behavior if both peptide types would show the same RT characteristics. *E*, retention time of pArg-peptides in relation to their pSer (*circle*), pThr (*triangle*), or pTyr (*square*) counterpart peptides. iRT peptides used for correlation of the RT are depicted in *purple*, and the linear regression based on the RT of iRT peptides is shown as *gray dashed line*. For all plots dots represent the average, and if applicable the standard deviation is displayed as error bars (n = 4).

Several groups tried to modify existing phosphopeptide enrichment strategies by using slightly less acidic environments, to make them more suitable for enriching the acid-labile arginine phosphorylation (8, 21). In order to demonstrate that pArg is actually stable under the acidic conditions that have been used for pHis enrichments (12) and to validate the identification of new protein-arginine phosphorylation in *S. aureus*, we analyzed 17 synthetic peptides representing randomly picked endogenous phosphopeptides identified in this study. First, we analyzed the chemical stability of arginine phosphorylation. Schmidt *et al.* (8) demonstrated that the phosphoramidate bond is rapidly hydrolyzed under acidic conditions (pH < 3). However, it was recently shown that the phosphoramidate bond of pHis is relatively stable under acidic conditions (pH 2.3, room temperature) (12). We analyzed the chemical stability of pArg under acidic conditions (pH 2) at 4 °C over a time course of 2 h (0, 15, 30, 60, 120 min). These conditions mimic the phosphopeptide enrichment conditions as well as loading conditions for the subsequent LC-MS/MS analysis. Under those conditions the synthetic pArg-peptides proved to be rather stable (Fig. 2*A* and supplemental Table S5). In order to see if the unmodified peptides became more predominant over time, the ratio of pArg-peptide to unmodified peptides over the time course of 2 h was determined (Fig. 2*B*). This demonstrated that all peptide ratios remain stable even after 2 h, indicating the stability of synthetic pArg under acidic conditions. Next, we incubated the pArg-peptides under acidic conditions for 24 h at room temperature. Only five pArg-peptides could be identified after 24 h, and for those a clear shift toward the unmodified peptide was observed (Fig. 2*C* and supplemental Table S5). These results convincingly show that the phosphoramidate bond of arginine is indeed acid labile but sufficiently stable to allow for phosphopeptide enrichment as well as LC-MS/MS analysis, similar to what has been shown for histidine phosphorylation.

We also analyzed the RT behavior of pArg-peptides in comparison with the unmodified peptides. pArg-peptides elute later than their respective unmodified counterpart peptides (Fig. 2*D* and supplemental Table S5). This is in line with previous studies showing that phosphorylation in general increases the RT in reversed phase chromatography (35, 36). Previously, we showed that pHis peptides elute predominantly later than their pSTY counterpart peptide (37). Unmodified histidine contributes the least to retention on C18 (35), and thus phosphorylation of histidine might increase the RT due to charge neutralization (36, 37). Unmodified arginine only contributes slightly more to the RT on C18 (35); therefore, we investigated if pArg is similar to pHis eluting predominantly later than their pSTY counterpart. This comparison showed a slight trend toward later elution (Fig. 2*E* and supplemental Table S5); however, this trend is not as strong as observed for pHis (37).

### HCD Outperforms ETD and EThcD in the Identification of Synthetic pArg-Peptides

Over the years different fragmentation strategies were developed that especially focus on the identification of labile phosphopeptides (27, 38). ETD fragmentation, which predominantly occurs along the backbone (N-Cα), is known to preserve labile phosphorylations; however, the fragmentation efficiency is highly dependent on the charge state, performing best for z ≥ 3 (27, 28). The problem of ETnoD events for lower charge states was solved by introducing EThcD that further improved the phosphorylation site localization compared with commonly used HCD fragmentation, which shows a prominent neutral loss (38, 39).

**Fig. 3:**
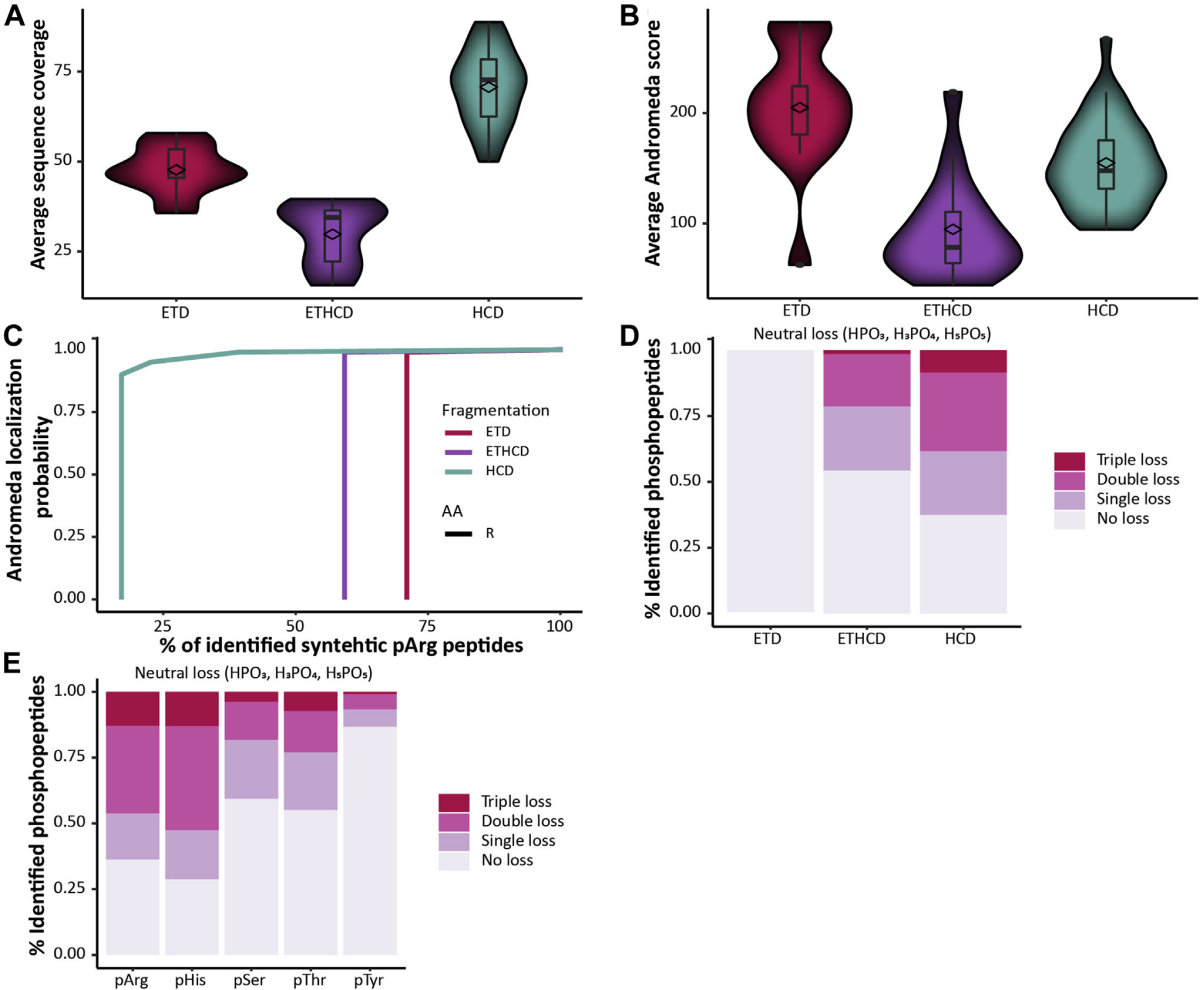
**Fragmentation characteristics of synthetic pArg-peptides.***A*, violin plot showing the average sequence coverage identified with ETD (*pink*), EThcD (*purple*), and HCD (*turquoise*) fragmentation. *B*, violin plot showing the average Andromeda score after ETD (*pink*), EThcD (*purple*), and HCD (*turquoise*) fragmentation. The mean values are highlighted as *diamond*. The median and first and third quantiles are depicted in the boxplot. Outliers are highlighted as individual points. *C*, Andromeda localization probability for the three fragmentation methods as function of the percentage of identified synthetic pArg-peptides. *D*, phosphate neutral loss triplets (*i.e.*, 80, 96, and 116 Da, respectively) observed in spectra derived from ETD, EThcD, and HCD fragmentation of synthetic pArg-peptides. Percentages of peptide-spectrum matches corresponding to synthetic pArg phosphopeptides that exhibit neutral losses. *E*, here, the percentages of peptide-spectrum matches corresponding to endogenous phosphopeptides identified in *S. aureus* samples that exhibit neutral losses are displayed for pSer, pThr, pTyr, pHis, and pArg-peptides.

Here, we compared HCD, ETD, and EThcD for the identification of synthetic pArg-peptides. A peptide mix combining all 17 synthetic peptides was analyzed using an Orbitrap Fusion with Orbitrap readout (n = 4). The acquired raw files were searched against a .fasta file containing all 17 synthetic peptides (see Experimental Procedures) and the resulting evidence.txt and msms.txt files were used to examine the sequence coverage and phosphosite localization. Taking all fragment ions contributing to the sequence identification into account, the average sequence coverage for the three different fragmentation methods was compared. Figure 3*A* shows that by far the highest sequence coverage was reached by HCD (70%) against 48% by ETD and only 30% by EThcD. In addition, in total 14 peptides were identified using HCD, whereas both the other techniques identified less than half of the synthetic pArg-peptides (ETD: 5, EThcD: 7, supplemental Fig. S7). To further assess the quality of the fragmentation and identification we compared the Andromeda scores, showing that ETD reaches the highest average Andromeda score with 204.9 (HCD: 154.5, EThcD: 94.7, Fig. 3*B*). These results are not in full agreement with previous studies comparing different fragmentation methods. Previous reports showed that collisional activation of ET products increased the sequence coverage compared with ETD or HCD alone especially for short, doubly protonated tryptic peptides (38, 40). Also, EThcD seems to be superior to HCD with respect to phosphosite assignment. For synthetic pArg-peptides the highest identification rate was previously observed by using ETD compared with HCD (29). ETD is known to perform significantly worse on peptides containing proline. The cleavage of the N-Cα bond keeps the resulting fragment attached to the remaining atoms of the ring, and therefore, c- and z-ions containing the N and C termini of proline are not observed (27). Indeed 7 of our 17 synthetic peptides contain at least one proline (supplemental Table S1). However, also peptides not containing any proline were not identified. Even though these findings are in contrast with commonly accepted notions, we are not the first ones seeing HCD outperforming especially ETD. A similar trend was observed when using a library of >100,000 unmodified and modified peptides (35). Also, in agreement with the work of Marx *et al.*, unambiguous localization (Andromeda localization probability of 1.0) of pArg was observed when using ETD and EThcD. However, as pointed out before, the number of identified pArg-peptides was low (Fig. 3*C* and supplemental Table S4). In comparison, HCD performed slightly worse with approximately 80% of all identified pArg-peptides being class I phosphosites (≥0.75) and 60% being unambiguously identified (Andromeda localization probability of 1.0). These findings underline the superiority of ETD and ETchD fragmentation in phosphosite localization as reported earlier (38, 40); however, the overall identification of our synthetic pArg-peptides was clearly hampered using ETD and EThcD compared with HCD. The superiority of phosphosite assignment has been shown to result from less phosphoric neutral losses when using ETD and ETchD (38, 40). Here, we can show that indeed no neutral loss was observed using ETD and that HCD proved to exhibit the highest amount of neutral loss (HPO_3_ = 79.966331 Da, H_3_PO_4_ = 97.976896 Da, and H_5_PO_5_ = 115.98746 Da, Fig. 3*D*). Using the endogenous identified phosphopeptides, we compared the amount of phosphoric neutral loss for the different amino acids (STYHR) and could show that the labile phosphoramidates (pHis and pArg) exhibit significantly more neutral loss, especially H_3_PO_4_ loss (Fig. 3*E*). This is in line with previous studies showing a significant neutral loss for pHis (37, 40) and pArg (29). Even though a triplet neutral loss is more characteristic for pHis and pArg, it does not represent unique evidence for the identification of phosphoramidates since only around 20% of PSMs matching pArg or pHis peptides showed the respective loss. Analyzing synthetic and endogenous pHis peptides, we could previously show the existence of a pHis-immonium ion that helps with the identification of the labile protein histidine phosphorylation (37). Accordingly, we also searched for respective pArg-immonium ions that could improve the localization. Indeed, we could identify two ions (237.0747 *m/z* and 209.0798 *m/z*) present in around 30% of MS2 spectra from synthetic pArg-peptides (supplemental Fig. S8). Searching MS2 spectra of synthetic pHSTY peptides revealed that those ions are not present in the respective MS2 spectra. However, the potential pArg-immonium ions were only present in less than 3% of MS2 from endogenous pArg-peptides. In comparison, 20% MS2 spectra from synthetic pHis peptides and 16.5% MS2 spectra from endogenous pHis peptides in *E. coli* showed the pHis-immonium ion of 190.0376 *m/z*.

### Synthetic Peptides Confirm Identification of Endogenous pArg

**Fig. 4:**
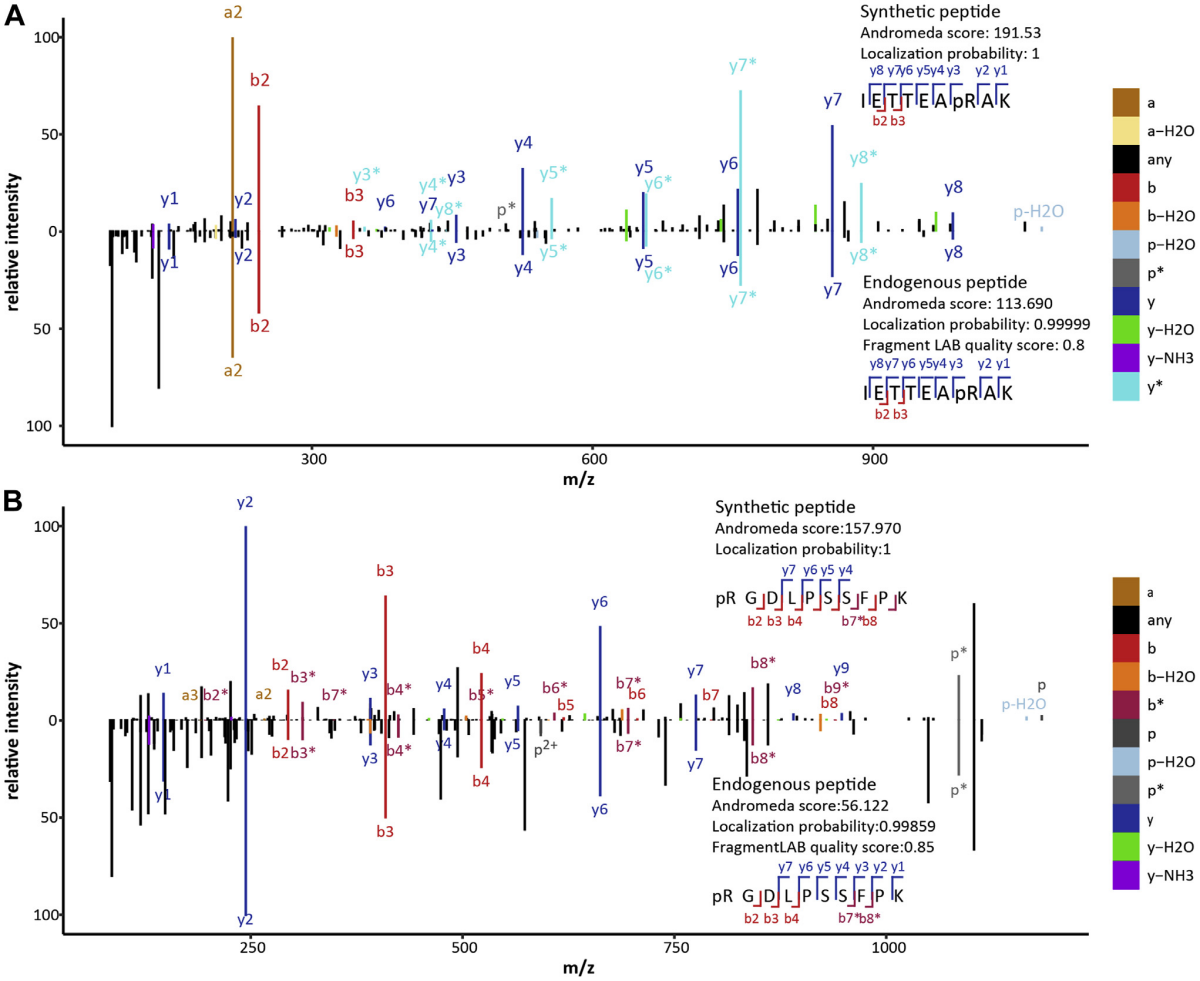
**Spectra comparison of synthetic and endogenous peptides.** Comparison of the acquired fragmentation spectra of synthetic and the respective endogenous peptides for (*A*) RplQ and (*B*) SAUSA300_2505. b- and y-ions are highlighted in *red* and *blue*.

To further validate endogenous pArg phosphorylation, we compared the acquired HCD spectra of our synthetic peptides with the spectra of our endogenous peptides (supplemental Fig. S9). Figure 4*A* shows the spectrum comparison for the 50S ribosomal protein L17 (RplQ). The endogenous peptide was identified with an Andromeda score of 113.69 and a localization probability of 0.99999. The synthetic peptides had an Andromeda score of 191.53 and a localization probability of 1. For both peptides almost the complete y-ion series (y1–y8) including y3-ion allowing the unambiguous localization of the phosphorylation site as well as two b-ions (b2 and b3) were identified showing a high similarity between the two spectra and therefore confirming the identification of pR36 on RplQ. For the Acetyltransferase SAUSA300_2505 the overlap of identified fragments was not as high as for RplQ; still we could show a high degree of similarity between the spectra of the synthetic and endogenous peptide (Fig. 4*B*). The localization of the phosphorylation site is high for both peptides (synthetic peptide: 1, endogenous peptide 0.99981). The Andromeda score of the synthetic peptides was 157.97, whereas the endogenous peptide only reached 56.122. The endogenous spectrum shows more peaks especially in the lower *m/z* range, which can simply be the result of coeluting peptides. Nevertheless, the identified b- and y-ions match perfectly to the fragment ions identified for the synthetic peptide and thus provide strong evidence for the accurate assignment of the spectrum. A peptide of the MutT/nudix family protein (Uniprot ID: A0A0H2XHE8) was identified to be phosphorylated on Thr as well as Arg. This allowed us to compare both spectra to the synthetic pArg-peptide, showing a clear deviation in the b4 ion for the endogenous pThr peptide (supplemental Fig. S10) compared with the synthetic pArg-peptide where all identified b- and y-ions for the endogenous pArg-peptide matched. We also compared the synthetic pThr peptide with the endogenous pThr peptide, which showed greater similarity than compared with the synthetic pArg-peptide (supplemental Fig. S9). This shows that the identification and phosphorylation site assignment of the endogenous peptides is highly accurate and clearly supports the high number of identified pArg-peptides in this study.

### Stp1 Is Not a Direct Arginine Phosphatase

**Fig. 5:**
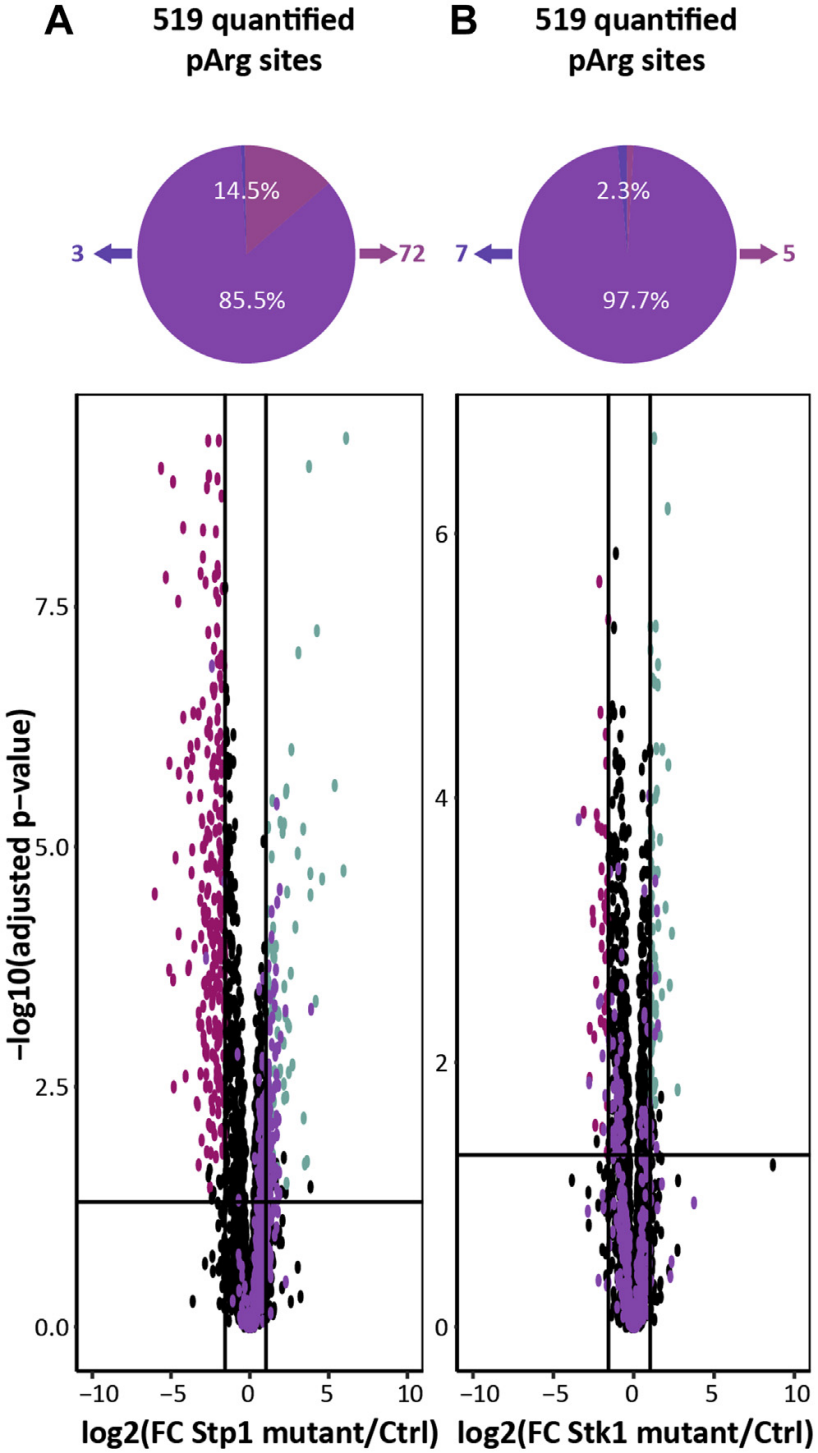
**Quantitative analysis of *S. aureus* mutants*****.**A*, pie chart showing the changes in pArg phosphorylation and volcano plot showing the quantitative changes in all phosphorylation sites between Stp1 mutant and Ctrl, identifying 411 changes (Tukey HSD *p*-value cutoff of 0.05 and a fold change cutoff of $\;\bar{x}\pm \;\sigma$ of the data). Quantified pArg sites are highlighted in *purple* and show a clear overrepresentation in the Stp1 mutant. Underrepresented p-sites are shown in *pink* and overrepresented sites in *turquoise*. Three pArg sites were underrepresented, and 72 pArg sites were overrepresented. *B*, pie chart showing the changes in pArg phosphorylation, and volcano plot comparing the phosphorylation sites between Stk1 mutant and Ctrl, identifying 117 changes (Tukey HSD *p*-value cutoff of 0.05 and a fold change cutoff of $\;\bar{x}\pm \;\sigma$ of the data). Seven pArg sites were underrepresented and five pArg sites overrepresented. All identified pArg sites are equally distributed between Stk1 mutant and Ctrl (*purple dots*).

Quantitative analysis of arginine phosphorylation in the three mutant strains revealed that 44 class I pArg sites were exclusively identified in the Stp1 mutant and 72 class I pArg sites were significantly overrepresented compared with the control (Tukey HSD *p*-value cutoff of 0.05 and a fold change cutoff of $\;\bar{x}\pm \;\sigma$ of the data) (supplemental Table S6) (Fig. 5*A*). Overall, all pArg-sites identified were skewed toward an overrepresentation in the Stp1 mutant compared with the control (Fig. 5*A*). At the same time pArg-sites were not shown to be regulated between the Stk1 mutant and the control (Fig. 5*B*). The majority of identified pArg-sites in the Stp1 mutant showed an overrepresentation compared with the control, whereas a true on/off regulation of target sites was previously reported for the ΔPtpB (arginine phosphatase) mutant (21). Still the overrepresentation in the Stp1 mutant provides strong evidence for a regulatory effect of Stp1 on the arginine phosphoproteome in *S. aureus* USA300.

To get more insight into the functional relevance of arginine phosphorylated proteins we performed a GO-term as well protein class enrichment using PANTHER (32) and compared it with Ser/Thr phosphorylated proteins (supplemental Fig. S11). GO terms related to protein transcription/translation as well as metabolic pathways were enriched according to a Fischer exact test. Protein-arginine phosphorylation has been shown to be involved in the general stress response (8, 23) as well as marking proteins for degradation within the ClpCP proteasome (34); therefore, it is also of no surprise that those terms were enriched. Next, we tried to identify sequence motifs surrounding pArg-sites. In line with previous studies on *B. subtilis* (8) and *S. aureus* (21) we could not identify a preferred sequence motif using pLogo (41) neither for sites solely identified in the Stp1 mutant nor for all identified pArg-sites (supplemental Fig. S12). This supports the hypothesis that protein-arginine kinases do not necessarily have a substrate specificity (8). It is hypothesized that arginine kinases require additional regulatory mechanisms to achieve substrate specificity such as temporal activation, cellular localization or protein–protein interactions (8). The clear presence of a specific pArg proteome supports this hypothesis.

**Fig. 6:**
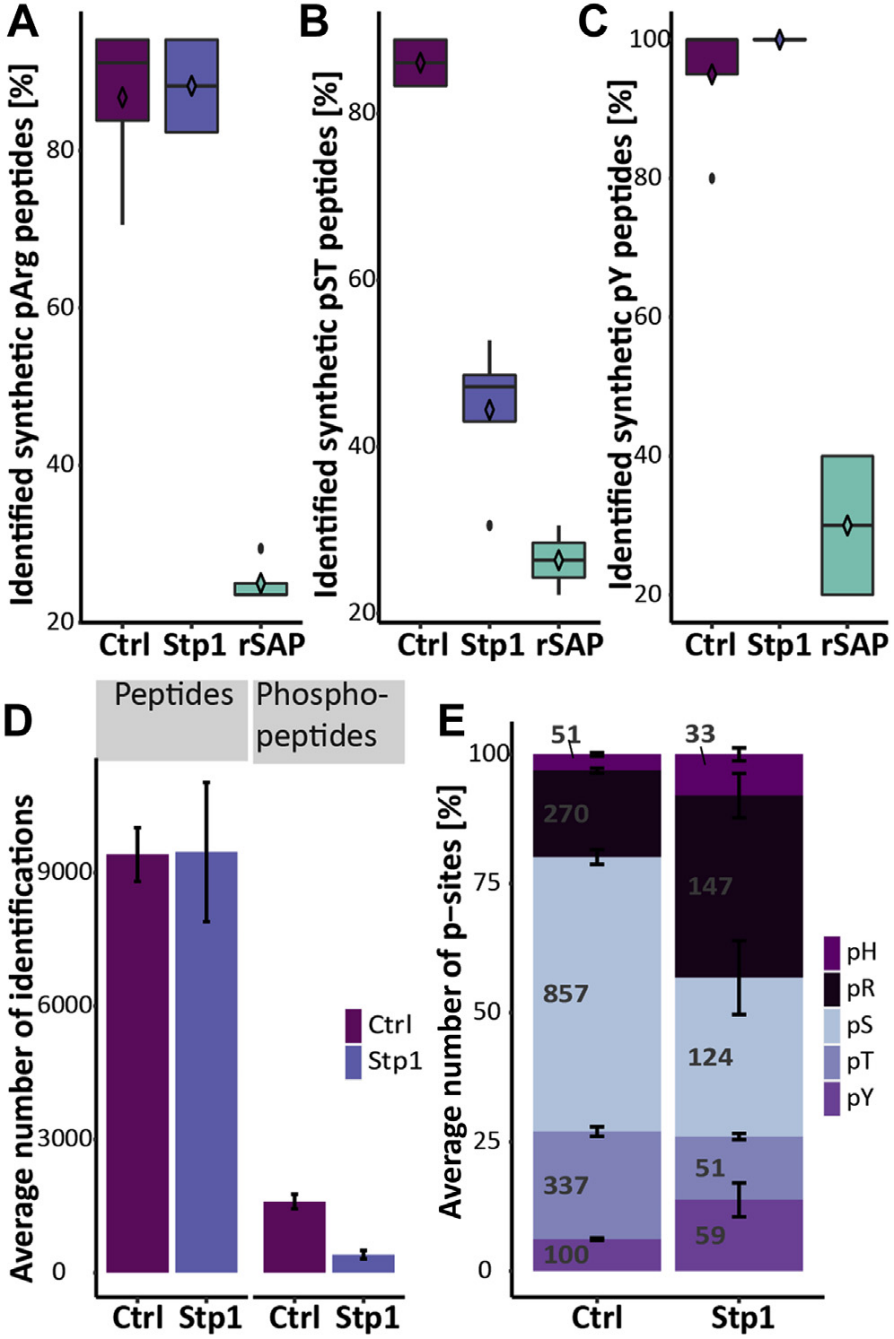
**Dephosphorylation of synthetic and endogenous phosphopeptides.** Percentage of identified synthetic (*A*) pArg, (*B*) pSer/Thr, and (*C*) pTyr after incubation without phosphatase, recombinant Stp1, or shrimp alkaline phosphatase rSAP (n = 4). *D*, average number of identified peptides and phosphopeptides after incubation with or without Stp1. *E*, distribution of serine, threonine, tyrosine, histidine, and arginine phosphosites identified. The stack bars represent the average percentage of phosphosites (n = 3) and the standard deviation is shown as error bars. *Numbers* depict the number of identified sites.

To investigate whether Stp1 has arginine phosphatase activity, we overexpressed and purified Stp1 (supplemental Fig. S13) and performed a dephosphorylation assay using synthetic pArg, pSer, pThr, or pTyr peptides as well as endogenous phosphopeptides. Although Stp1 did not show any influence on arginine or tyrosine phosphorylation (Fig. 6, *A* and *C*), incubation of Stp1 with pST peptides led to a decrease of identified synthetic pST peptides by almost 44% (Fig. 6*B*). As a positive control the shrimp alkaline phosphatase (rSAP) showed a decrease of identified phosphopeptide by up to 60% for all three types of phosphopeptides. Stp1 treatment also resulted in a decrease in phosphorylation after incubation with endogenous phosphopeptide, whereas the total number of identified peptides remained the same (Fig. 6*D*). Even though in this case phosphorylation generally decreased after treatment with Stp1, a clear preference for pS and pT dephosphorylation was observed (Fig. 6*E*). These results indicate that Stp1 does not have specific arginine phosphatase activity but rather has a secondary effect on the arginine phosphoproteome.

## Conclusions

Here we report the largest arginine phosphoproteome for *S. aureus* USA300 to date. In the future this dataset will help to shed light on the biological role of arginine phosphorylation that thus far is limited to a general stress response and marking proteins for degradation (8, 23, 34). Using synthetic pArg-peptides we were able to confirm that arginine phosphorylation is stable enough to be enriched under conditions suitable for histidine phosphorylation (12). Also, the extensive endogenous arginine phosphorylation was confirmed by comparing MS2 spectra to the synthetic counterparts supporting the confident phosphorylation site assignment. Using synthetic peptides we also showed that HCD is still the gold standard in phosphoproteomics and outperforms ETD as well as EthcD for the identification of 17 synthetic arginine phosphorylated peptides, whereas site assignment was comparable between the three fragmentation methods. Finally, we present strong evidence that the eukaryotic-like Ser/Thr phosphatase Stp1, even though not a direct pArg phosphatase, influences the *S. aureus* USA300 arginine phosphoproteome and thereby introduces an additional regulatory mechanism for arginine phosphorylation next to McsB and PtpB.

## Data Availability

All raw data that support the findings of this study have been deposited in ProteomeXchange with the accession number PXD026981.

## Supplemental data

This article contains supplemental data (41).

## Conflict of interest

The authors declare no competing interests.
