# Supplementary material for: Widespread Arginine Phosphorylation in Staphylococcus aureus
Source: Mol Cell Proteomics. 2022 Apr 12;21(5):100232. doi: 10.1016/j.mcpro.2022.100232 (PMC9112008; doi:10.1016/j.mcpro.2022.100232)
Supplement: Supplemental Figures S1–S13 [file mmc1.pptx]

## Slide 1
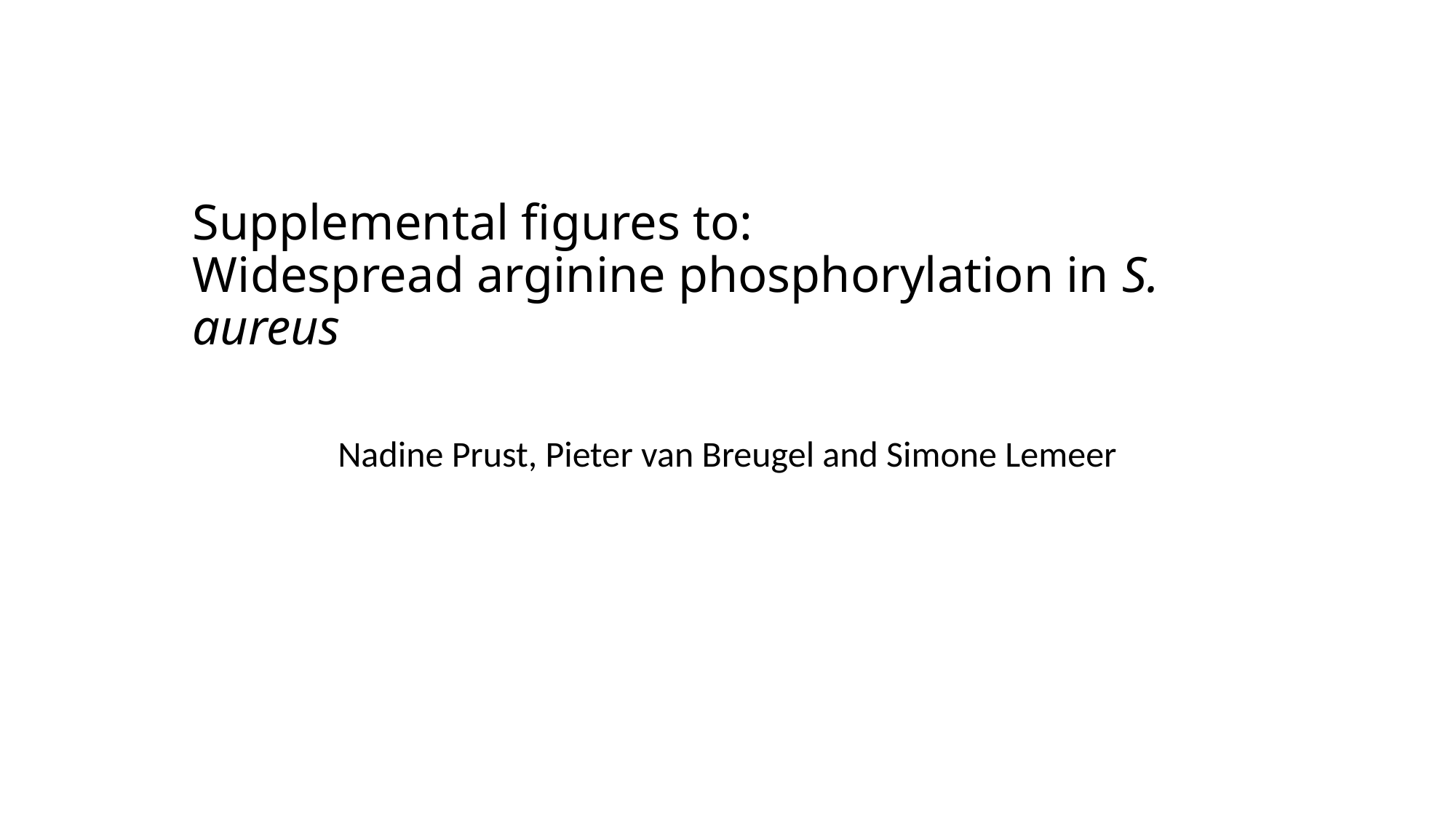

# Supplemental figures to: Widespread arginine phosphorylation in S. aureus
Nadine Prust, Pieter van Breugel and Simone Lemeer

## Slide 2
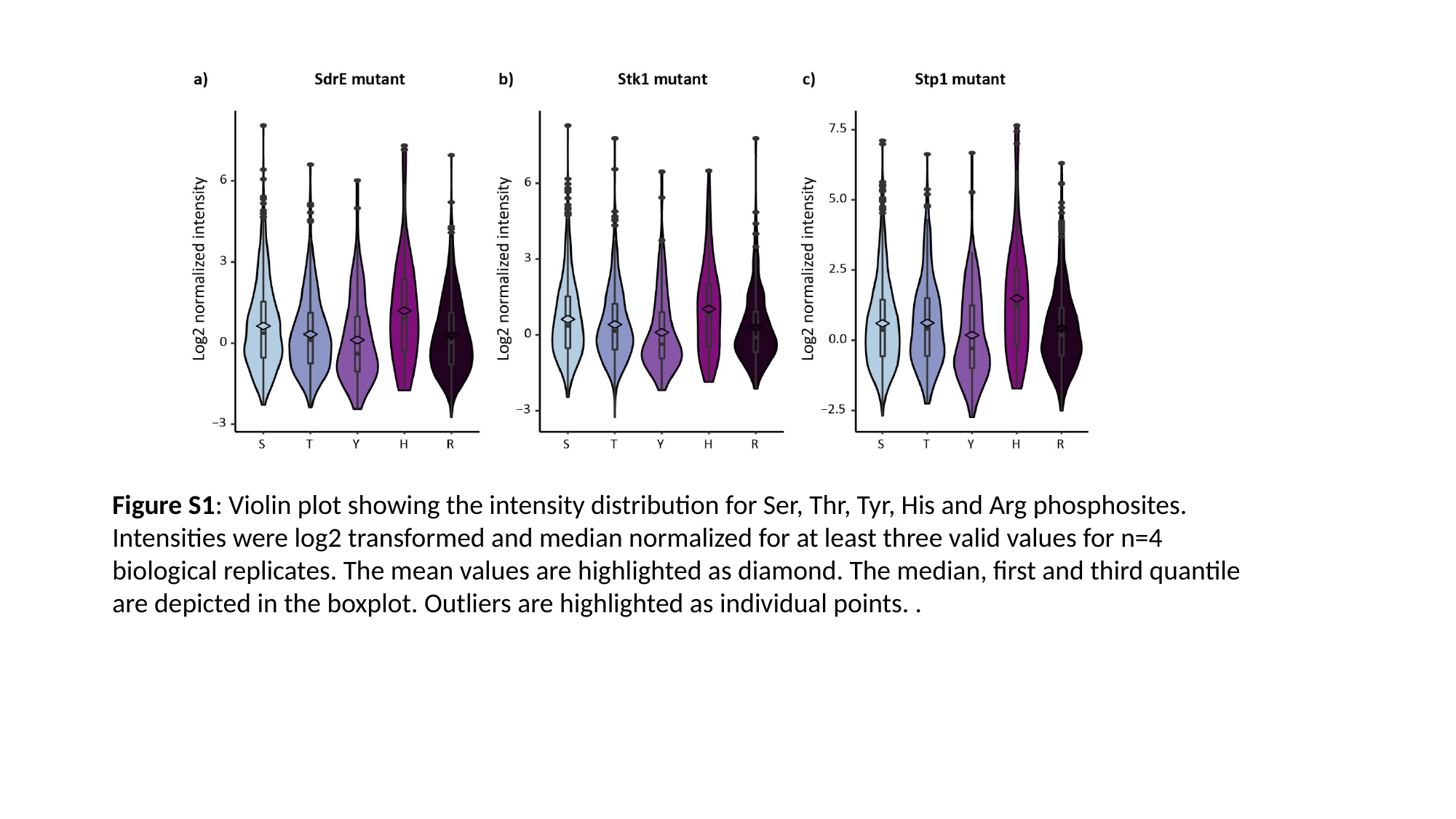

Figure S1: Violin plot showing the intensity distribution for Ser, Thr, Tyr, His and Arg phosphosites. Intensities were log2 transformed and median normalized for at least three valid values for n=4 biological replicates. The mean values are highlighted as diamond. The median, first and third quantile are depicted in the boxplot. Outliers are highlighted as individual points. .

## Slide 3
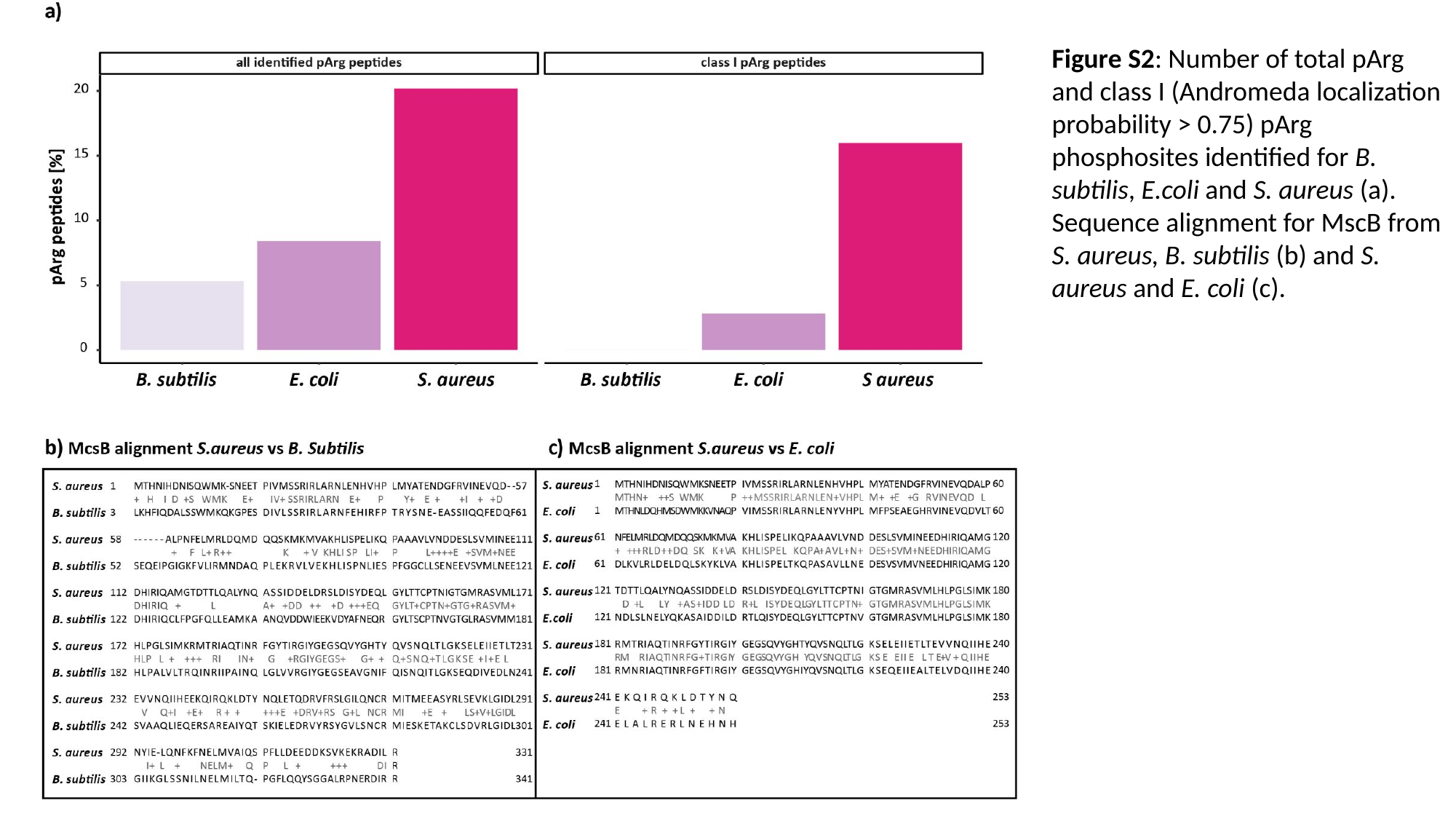

Figure S2: Number of total pArg and class I (Andromeda localization probability > 0.75) pArg phosphosites identified for B. subtilis, E.coli and S. aureus (a).
Sequence alignment for MscB from S. aureus, B. subtilis (b) and S. aureus and E. coli (c).

## Slide 4
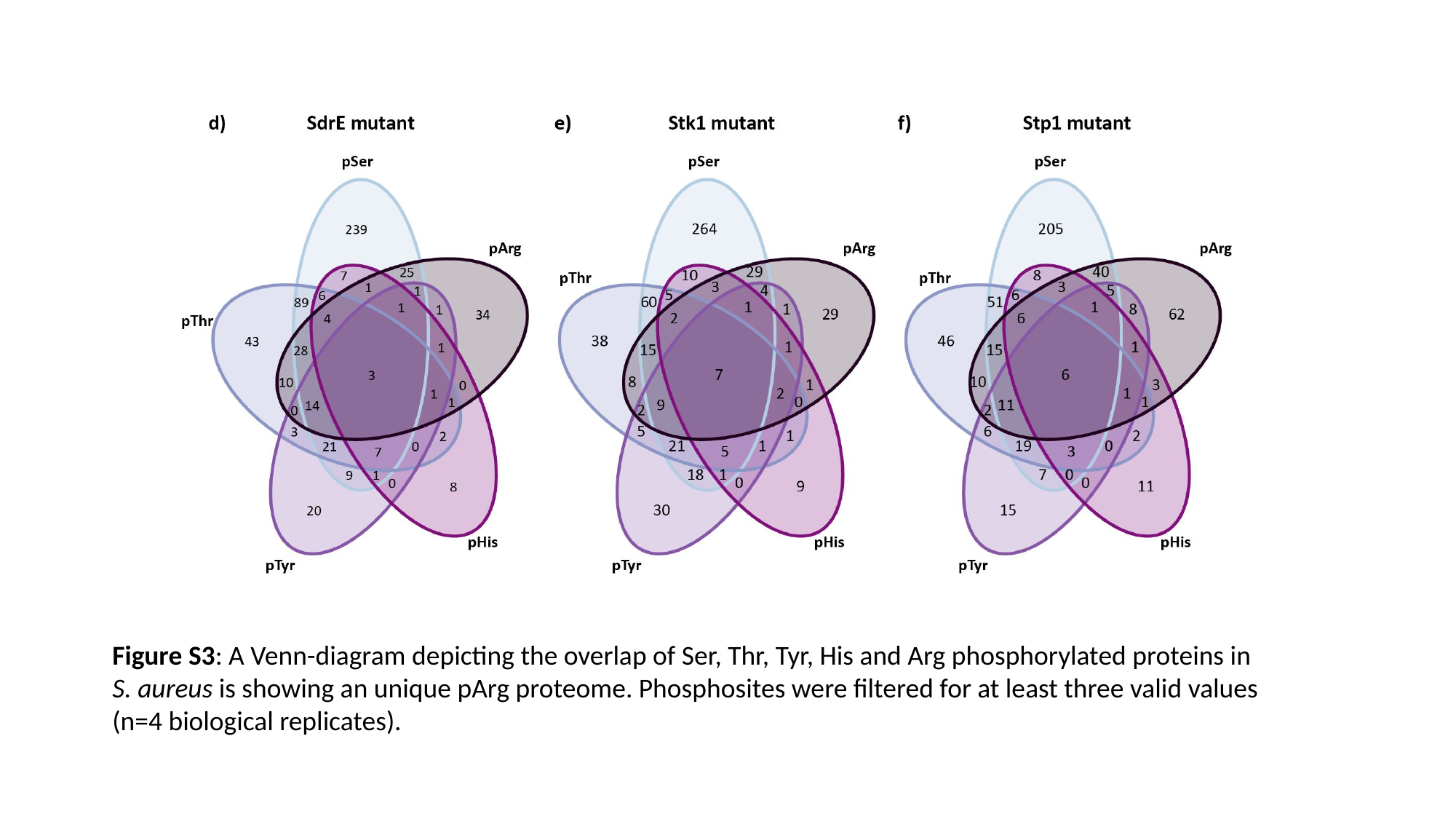

Figure S3: A Venn-diagram depicting the overlap of Ser, Thr, Tyr, His and Arg phosphorylated proteins in S. aureus is showing an unique pArg proteome. Phosphosites were filtered for at least three valid values (n=4 biological replicates).

## Slide 5
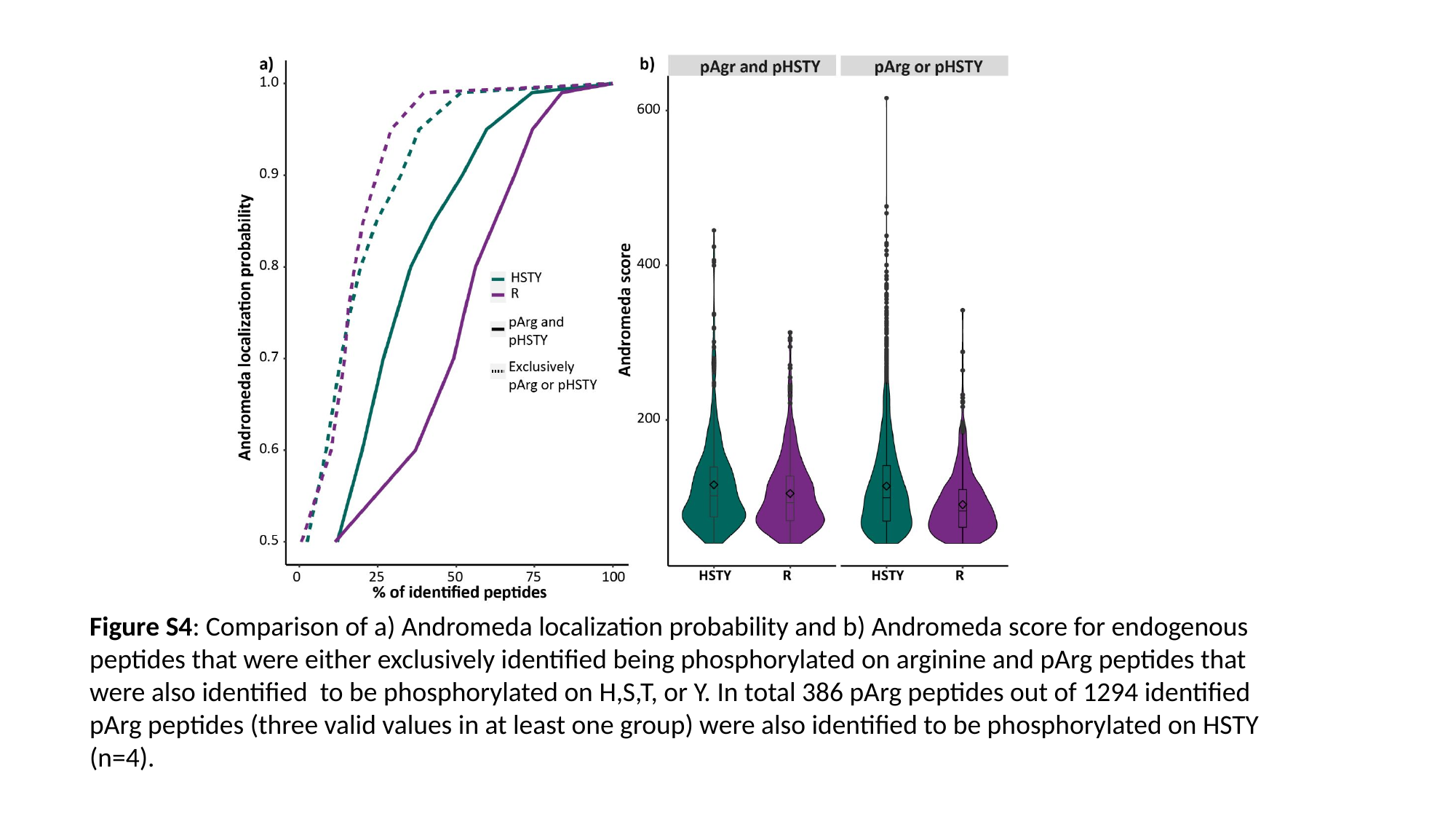

Figure S4: Comparison of a) Andromeda localization probability and b) Andromeda score for endogenous peptides that were either exclusively identified being phosphorylated on arginine and pArg peptides that were also identified to be phosphorylated on H,S,T, or Y. In total 386 pArg peptides out of 1294 identified pArg peptides (three valid values in at least one group) were also identified to be phosphorylated on HSTY (n=4).

## Slide 6
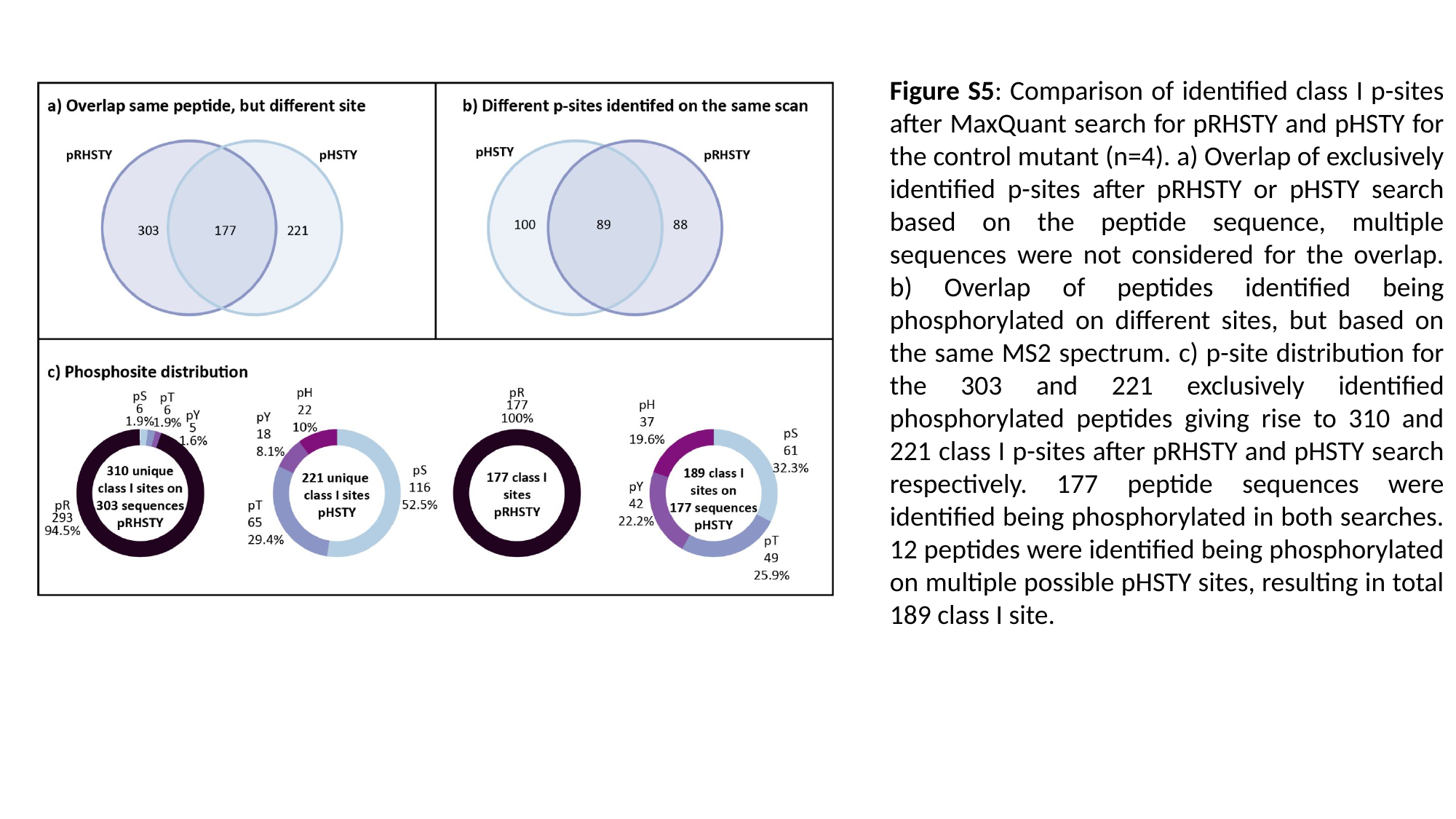

Figure S5: Comparison of identified class I p-sites after MaxQuant search for pRHSTY and pHSTY for the control mutant (n=4). a) Overlap of exclusively identified p-sites after pRHSTY or pHSTY search based on the peptide sequence, multiple sequences were not considered for the overlap. b) Overlap of peptides identified being phosphorylated on different sites, but based on the same MS2 spectrum. c) p-site distribution for the 303 and 221 exclusively identified phosphorylated peptides giving rise to 310 and 221 class I p-sites after pRHSTY and pHSTY search respectively. 177 peptide sequences were identified being phosphorylated in both searches. 12 peptides were identified being phosphorylated on multiple possible pHSTY sites, resulting in total 189 class I site.

## Slide 7
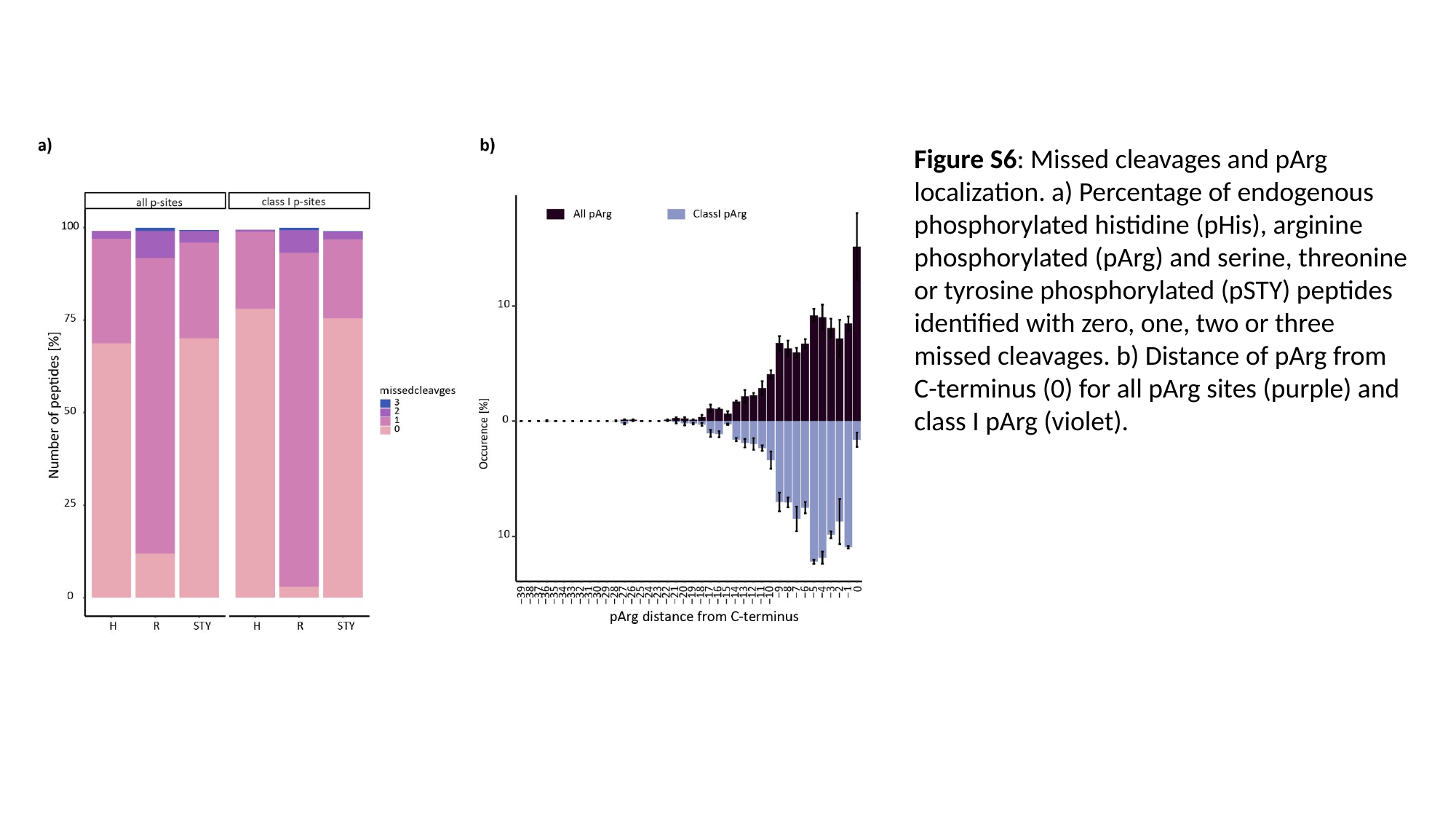

Figure S6: Missed cleavages and pArg localization. a) Percentage of endogenous phosphorylated histidine (pHis), arginine phosphorylated (pArg) and serine, threonine or tyrosine phosphorylated (pSTY) peptides identified with zero, one, two or three missed cleavages. b) Distance of pArg from C-terminus (0) for all pArg sites (purple) and class I pArg (violet).

## Slide 8
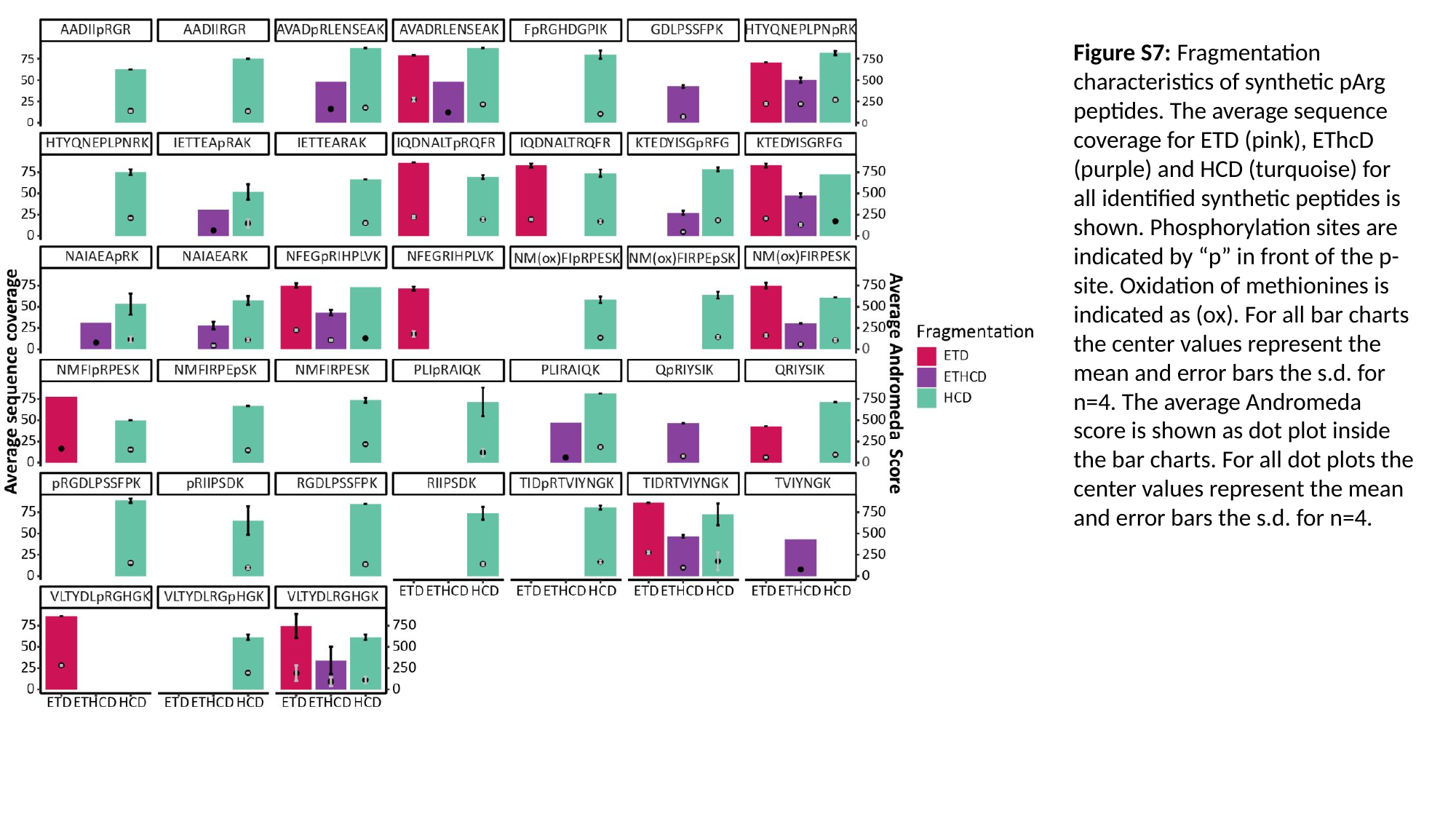

Figure S7: Fragmentation characteristics of synthetic pArg peptides. The average sequence coverage for ETD (pink), EThcD (purple) and HCD (turquoise) for all identified synthetic peptides is shown. Phosphorylation sites are indicated by “p” in front of the p-site. Oxidation of methionines is indicated as (ox). For all bar charts the center values represent the mean and error bars the s.d. for n=4. The average Andromeda score is shown as dot plot inside the bar charts. For all dot plots the center values represent the mean and error bars the s.d. for n=4.

## Slide 9
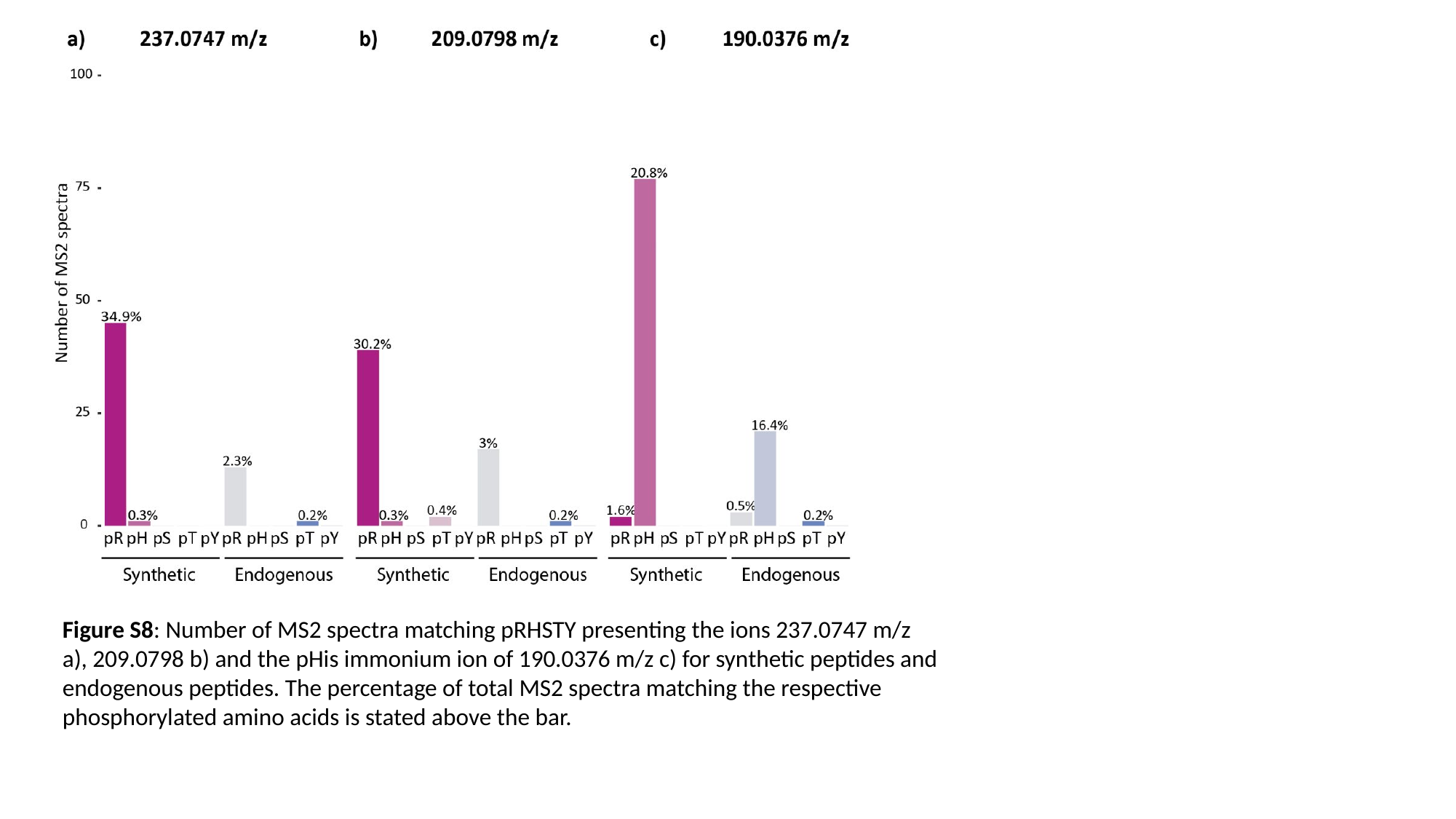

Figure S8: Number of MS2 spectra matching pRHSTY presenting the ions 237.0747 m/z a), 209.0798 b) and the pHis immonium ion of 190.0376 m/z c) for synthetic peptides and endogenous peptides. The percentage of total MS2 spectra matching the respective phosphorylated amino acids is stated above the bar.

## Slide 10
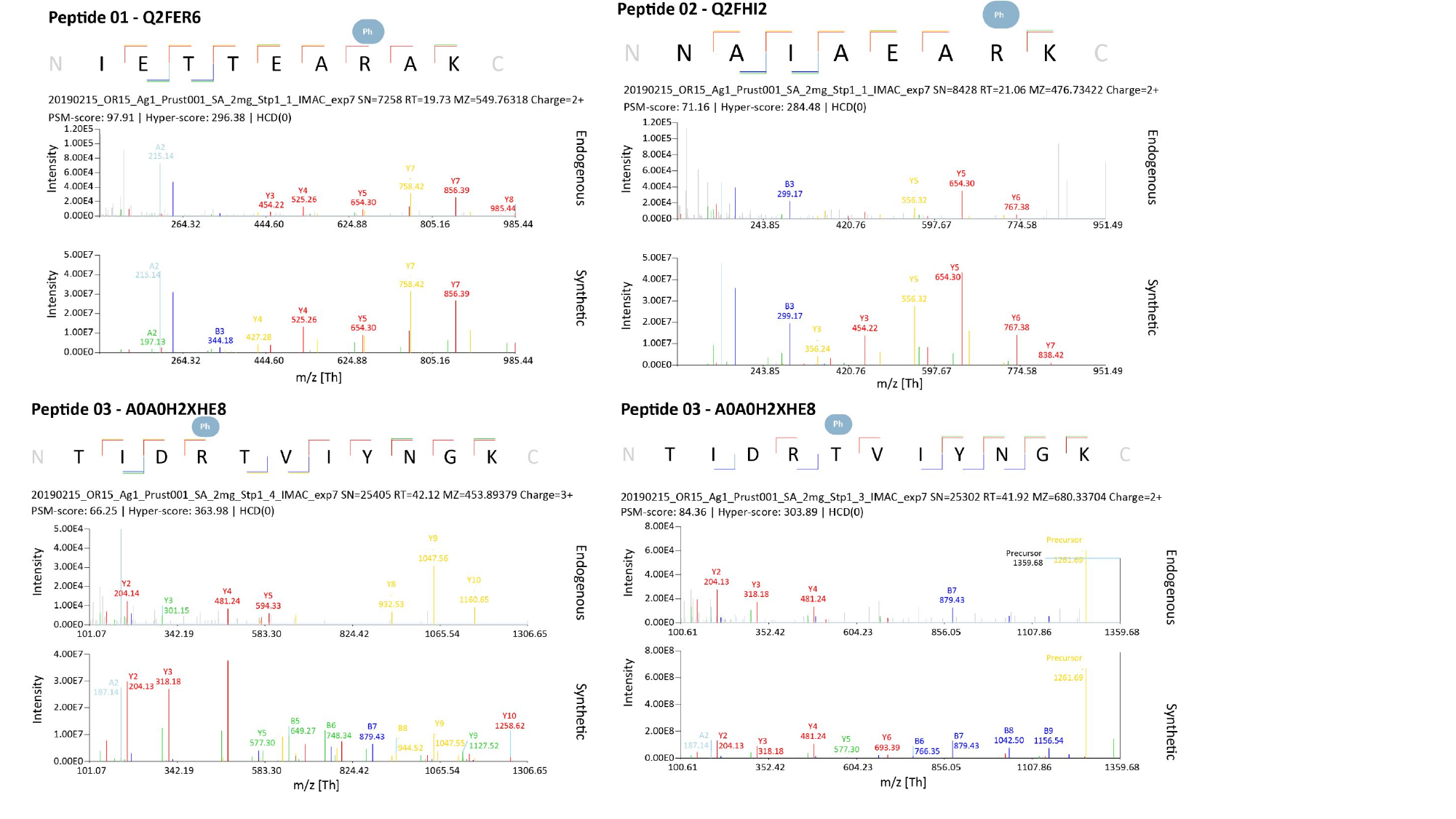

## Slide 11
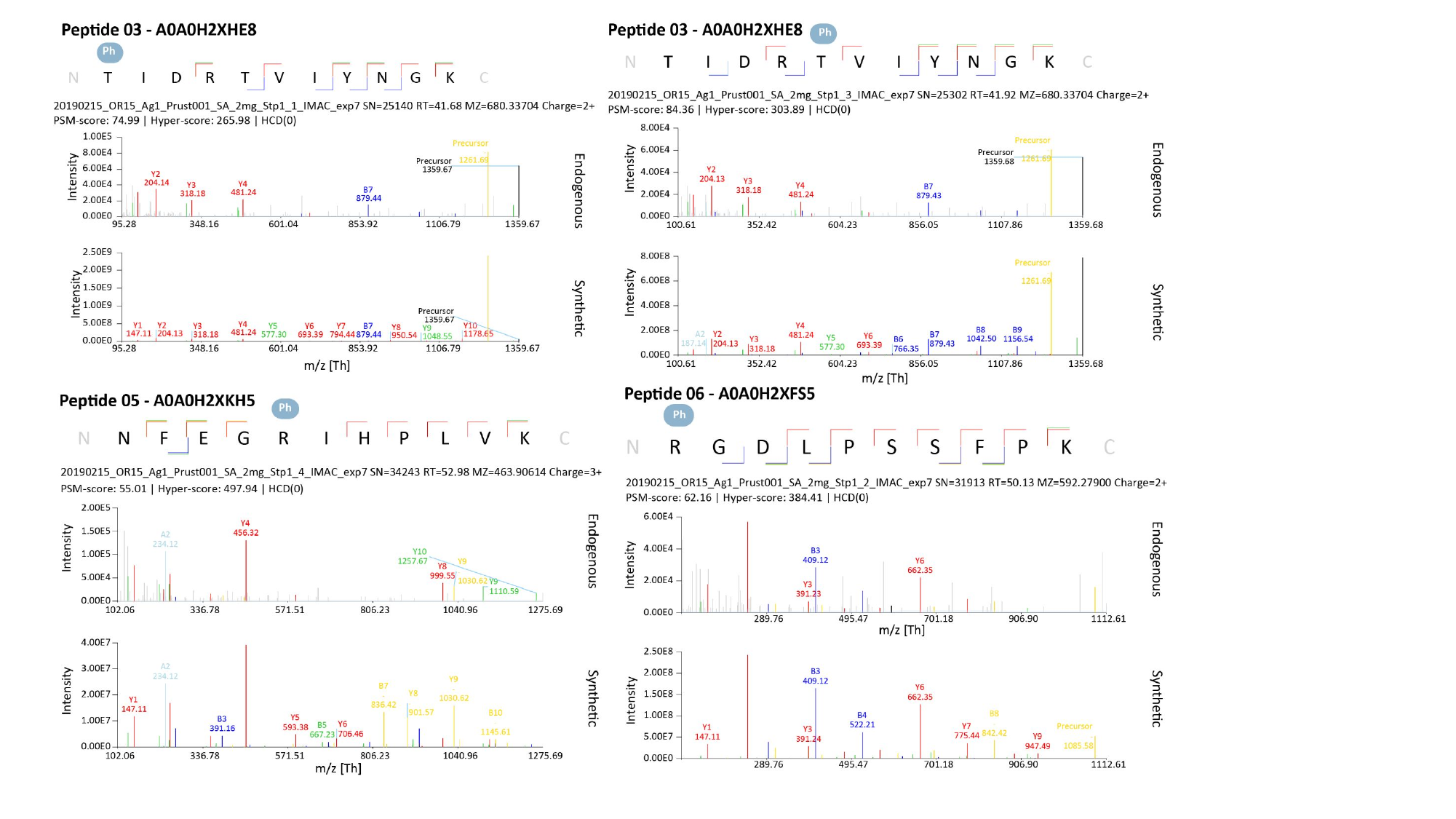

## Slide 12
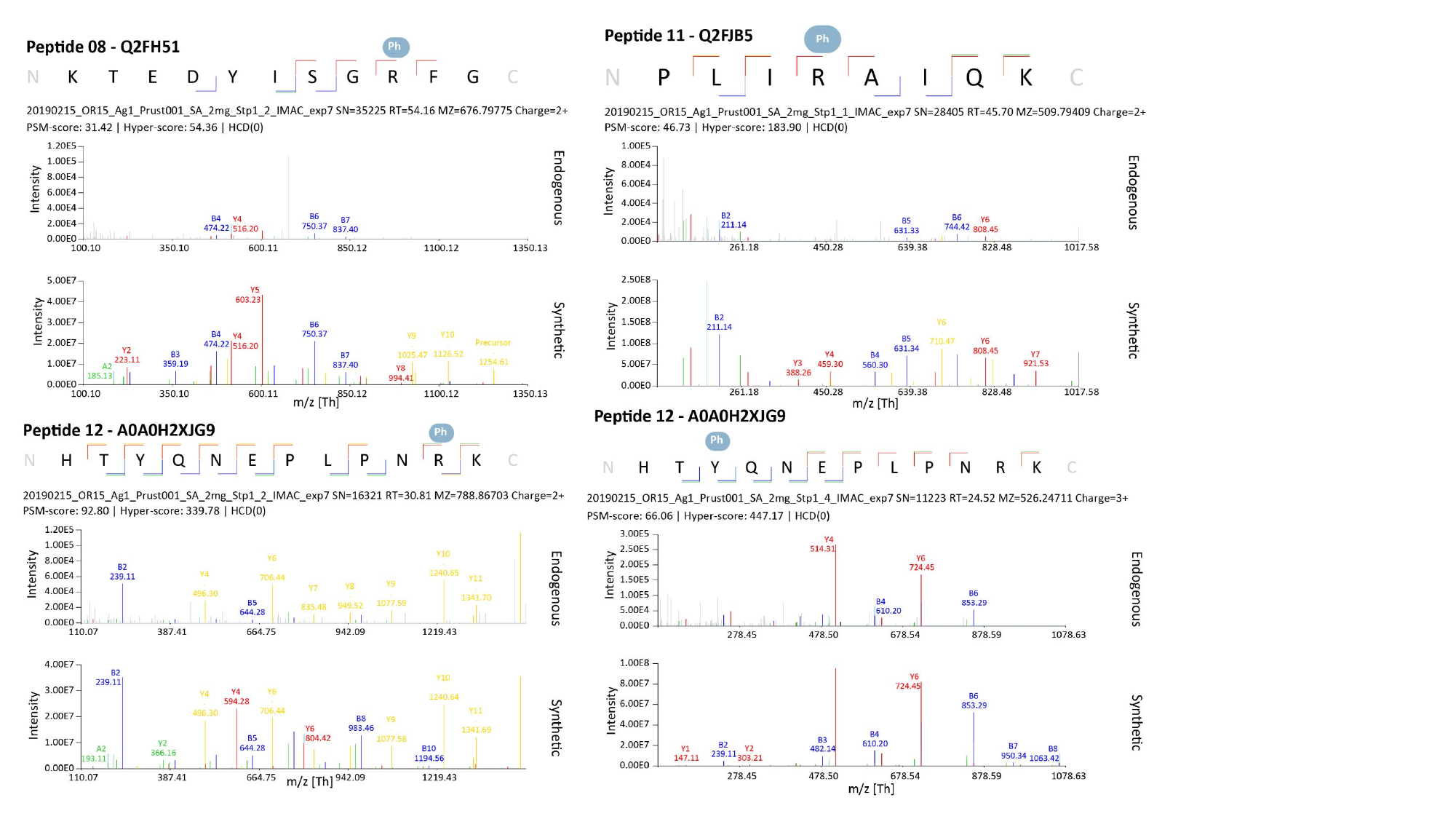

## Slide 13
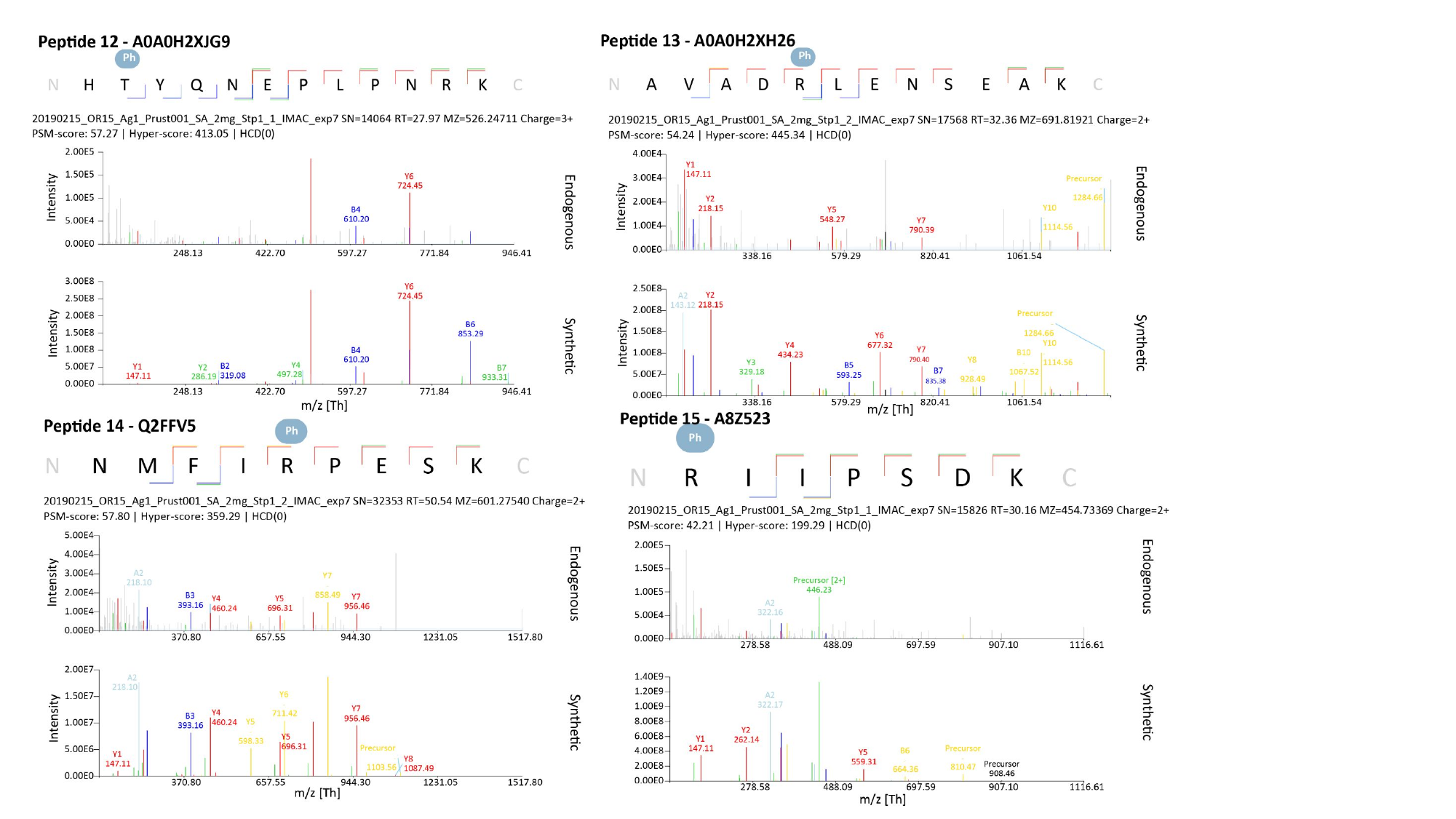

## Slide 14
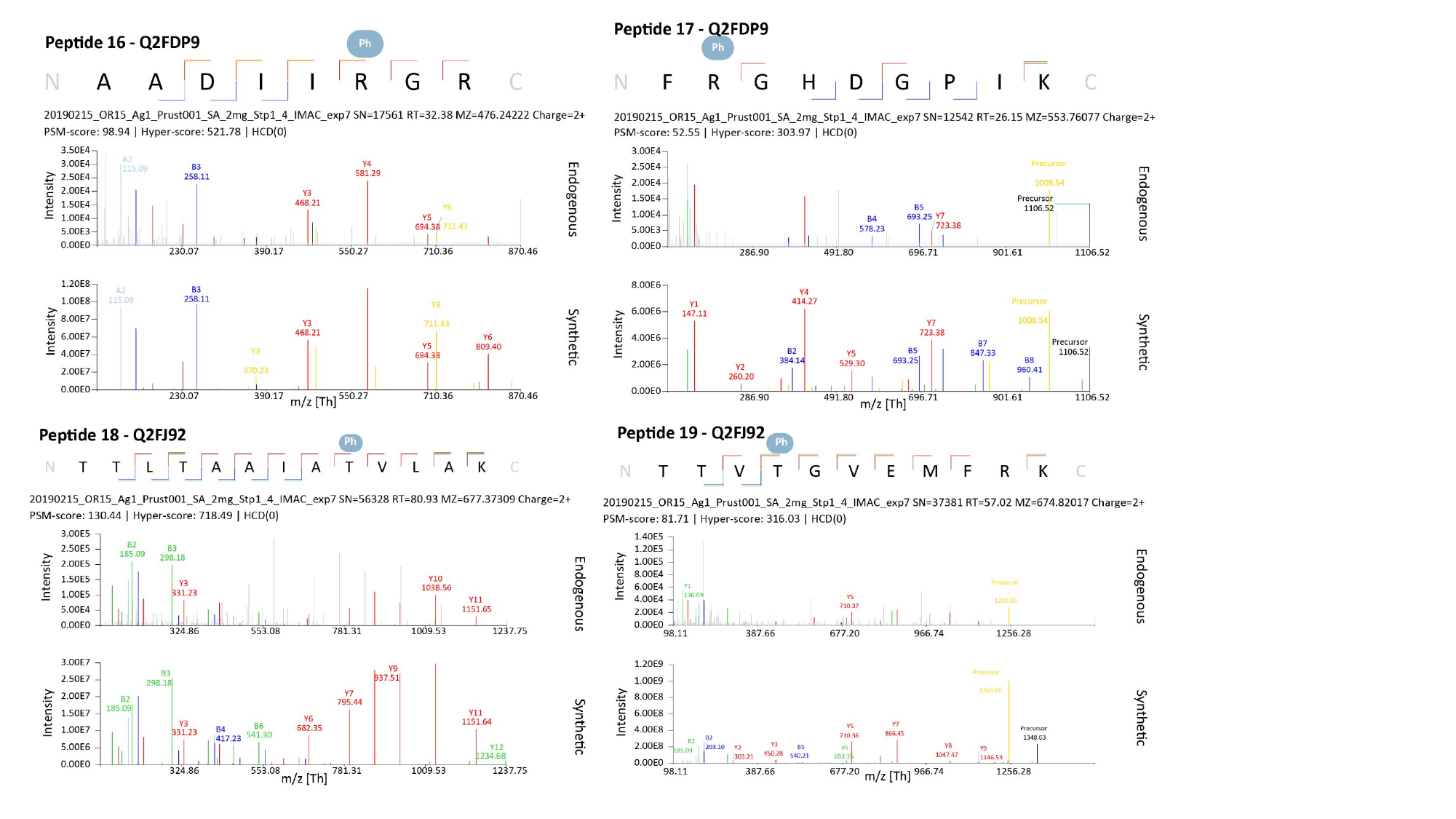

## Slide 15
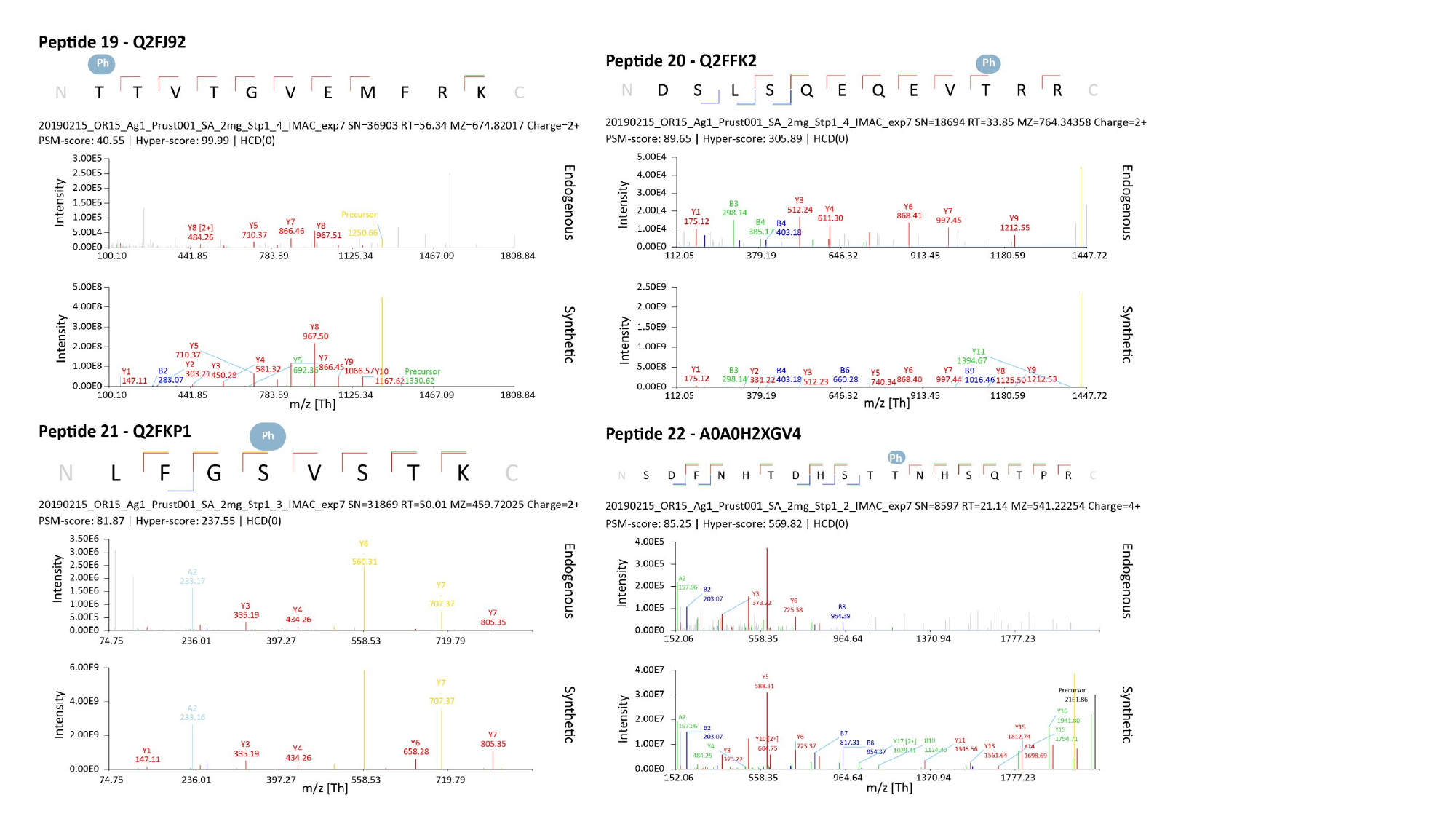

## Slide 16
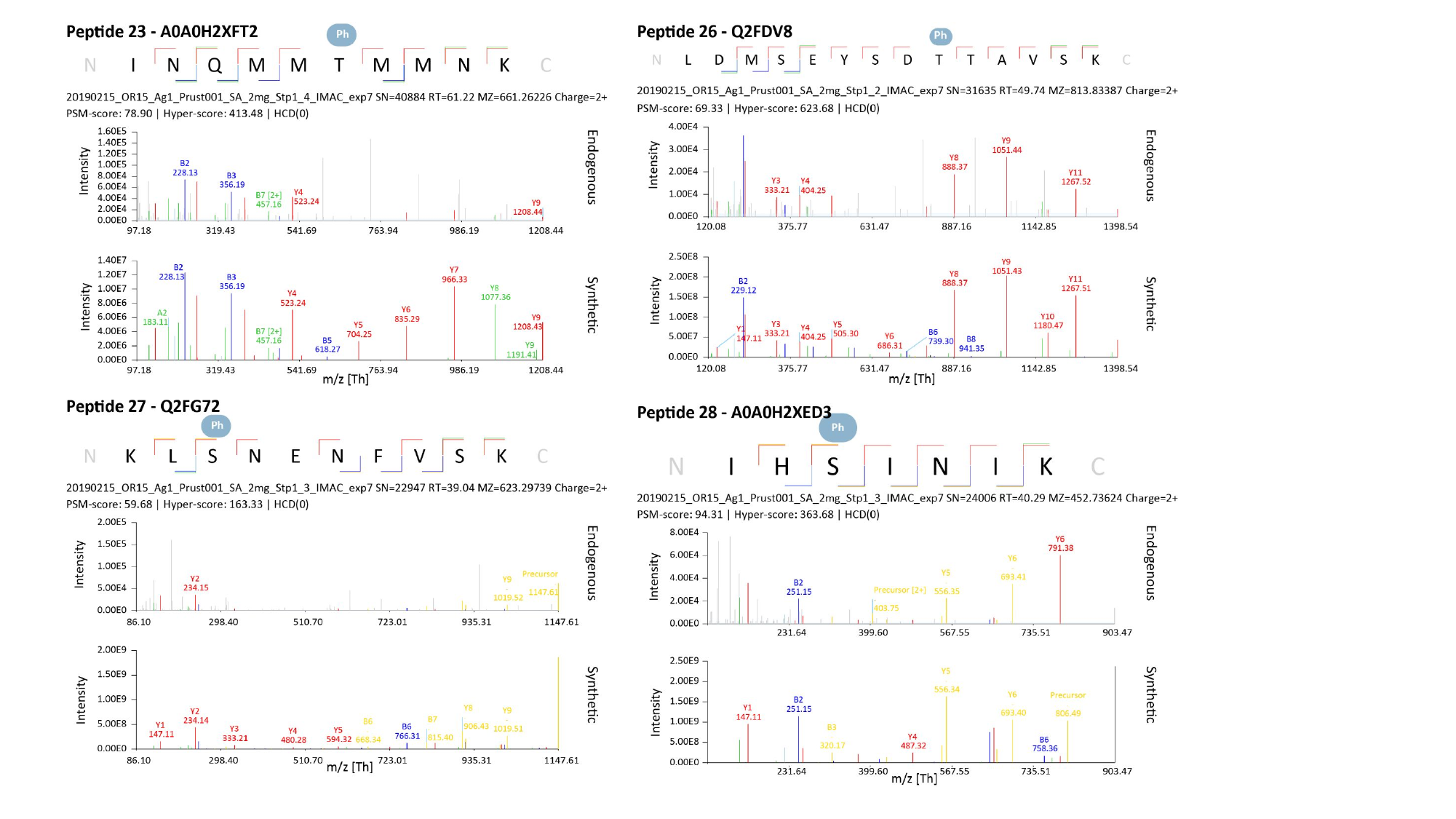

## Slide 17
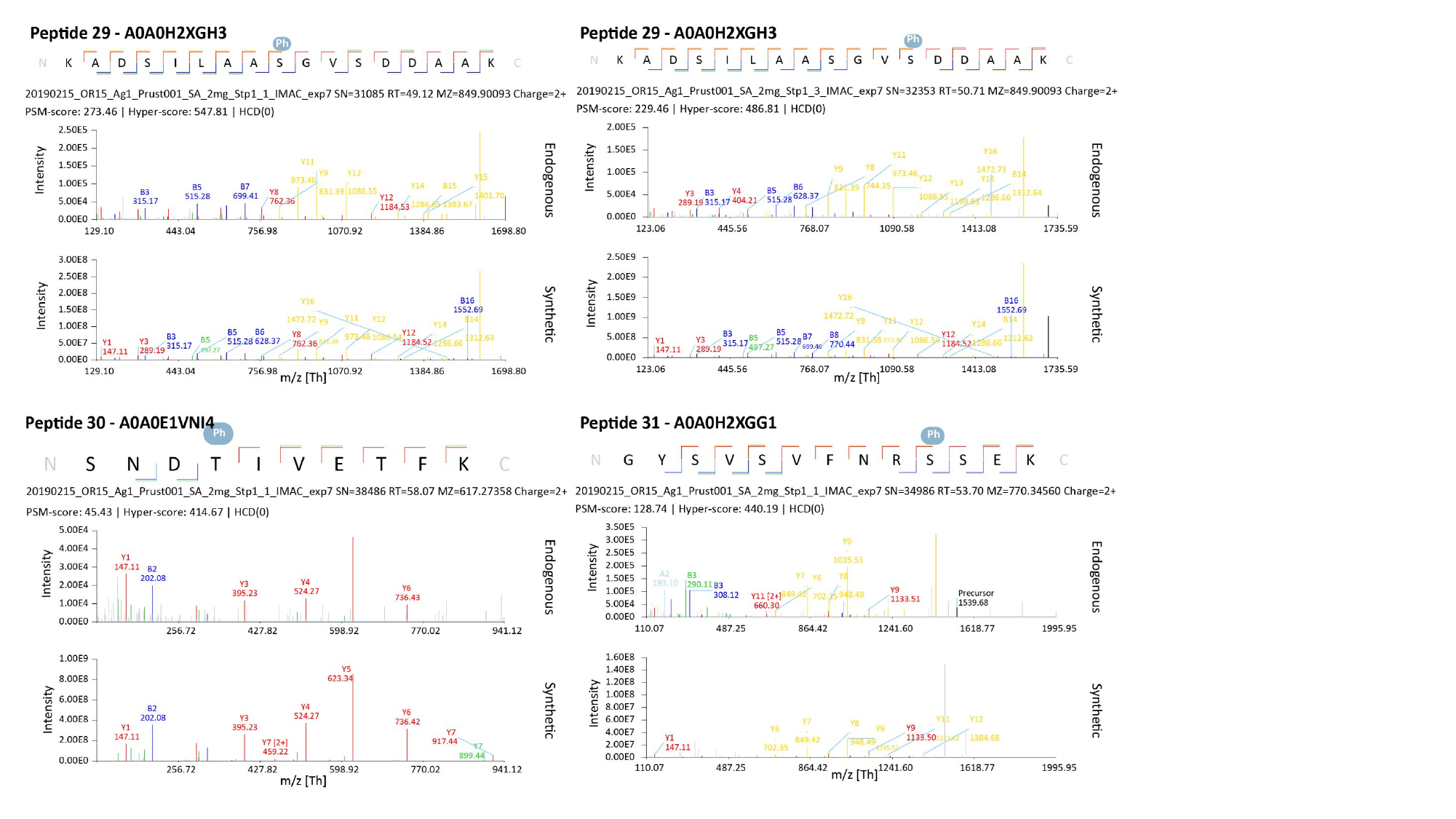

## Slide 18
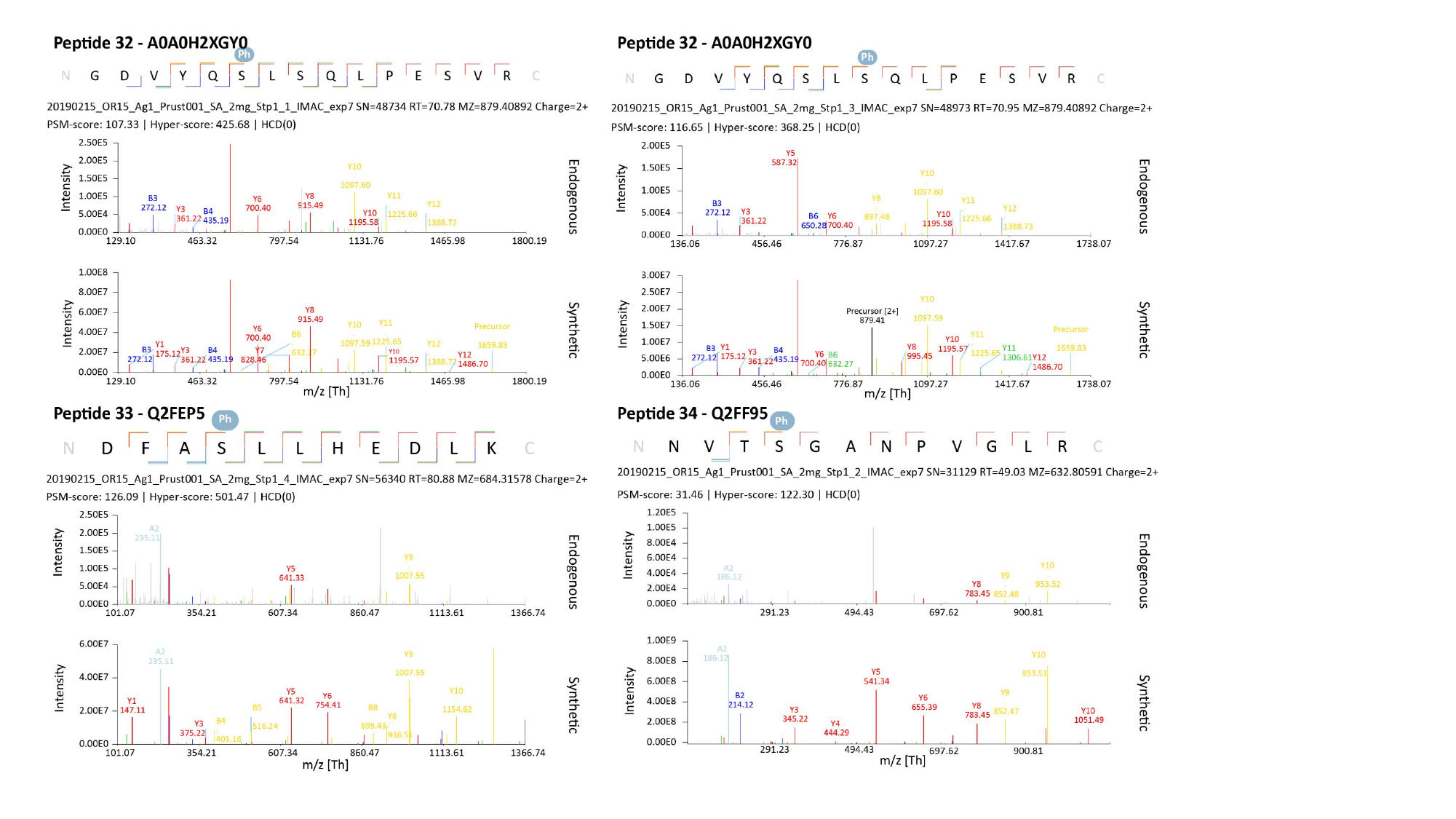

## Slide 19
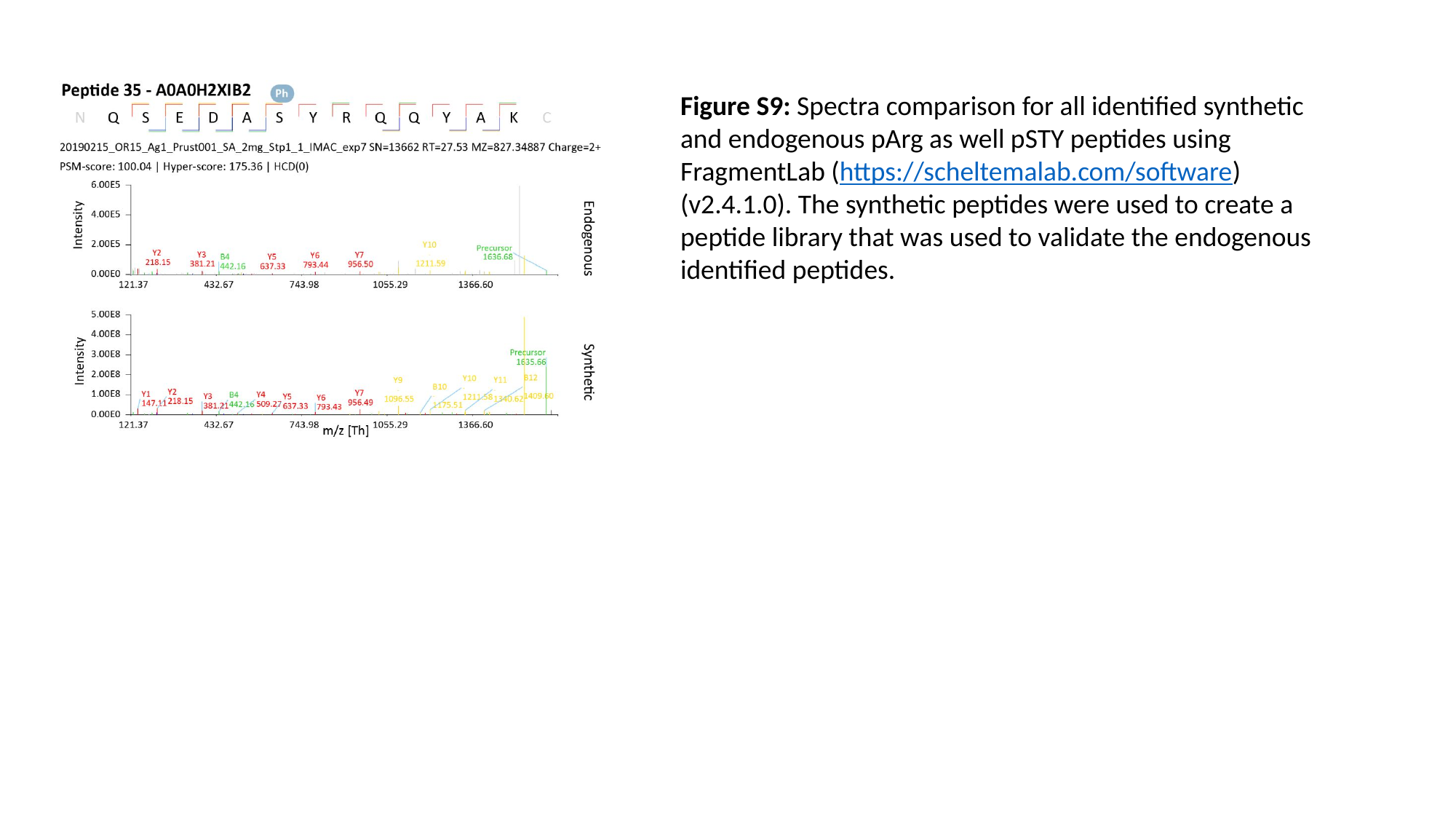

Figure S9: Spectra comparison for all identified synthetic and endogenous pArg as well pSTY peptides using FragmentLab (https://scheltemalab.com/software) (v2.4.1.0). The synthetic peptides were used to create a peptide library that was used to validate the endogenous identified peptides.

## Slide 20
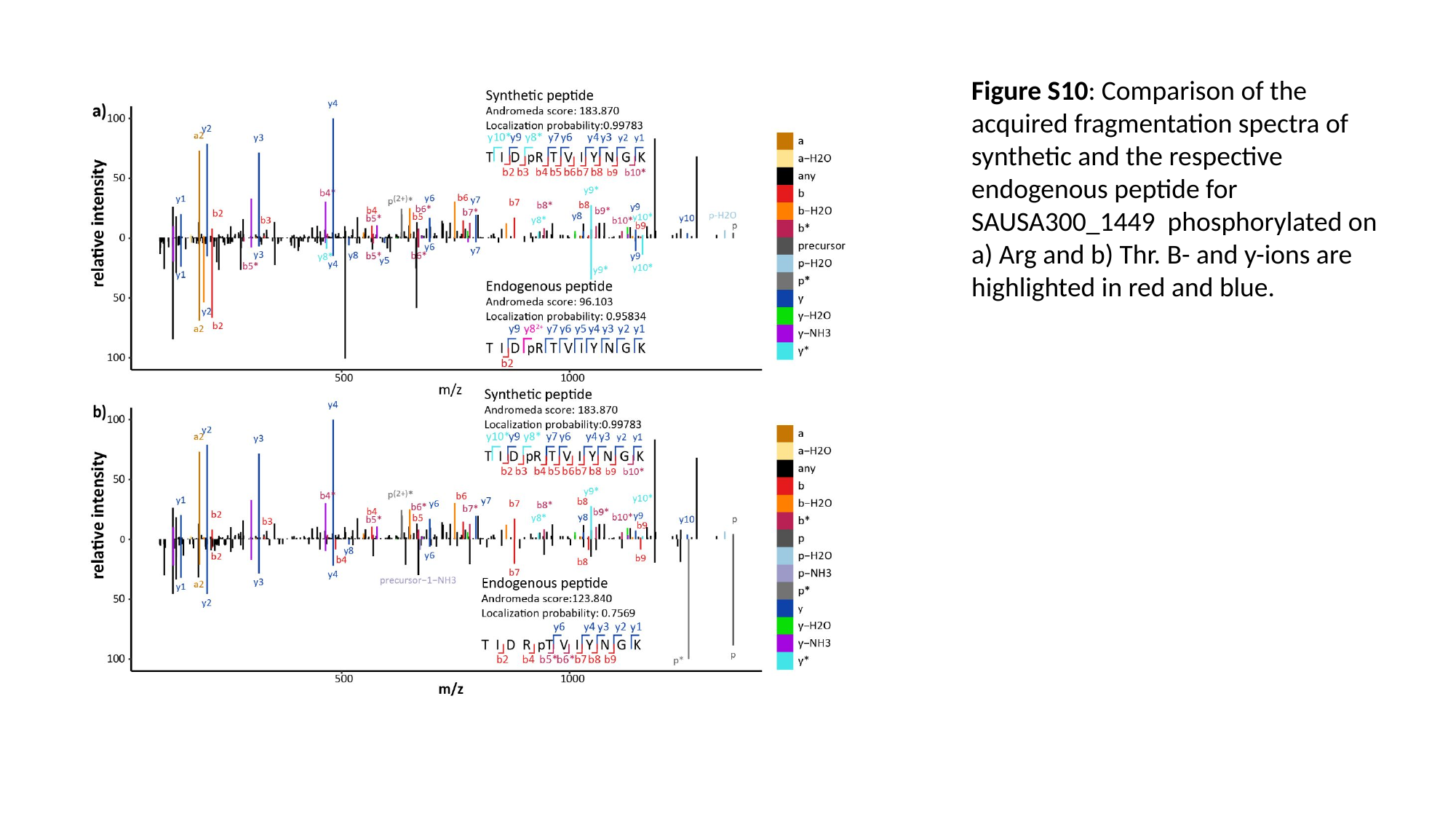

Figure S10: Comparison of the acquired fragmentation spectra of synthetic and the respective endogenous peptide for SAUSA300_1449 phosphorylated on a) Arg and b) Thr. B- and y-ions are highlighted in red and blue.

## Slide 21
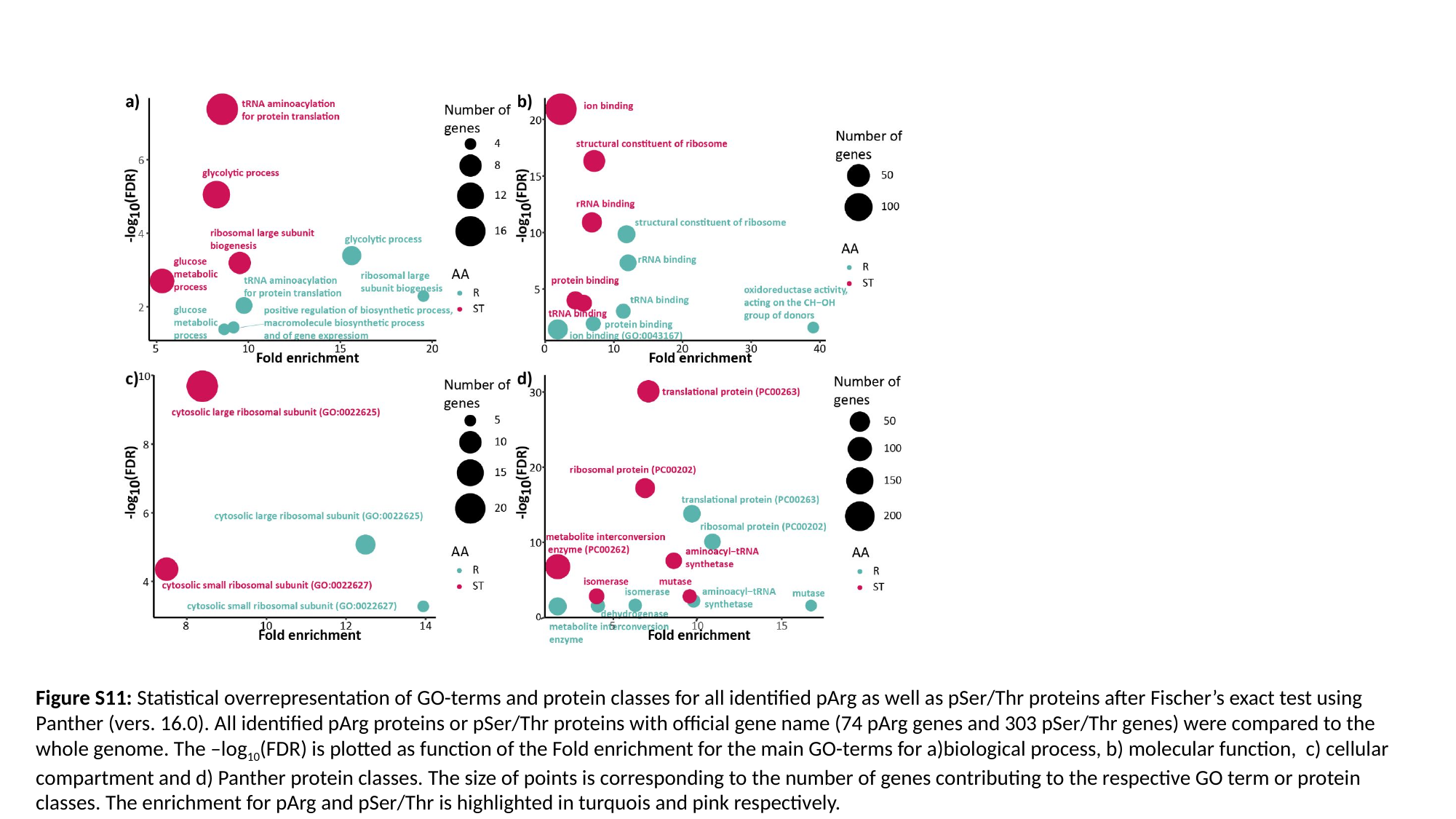

Figure S11: Statistical overrepresentation of GO-terms and protein classes for all identified pArg as well as pSer/Thr proteins after Fischer’s exact test using Panther (vers. 16.0). All identified pArg proteins or pSer/Thr proteins with official gene name (74 pArg genes and 303 pSer/Thr genes) were compared to the whole genome. The –log10(FDR) is plotted as function of the Fold enrichment for the main GO-terms for a)biological process, b) molecular function, c) cellular compartment and d) Panther protein classes. The size of points is corresponding to the number of genes contributing to the respective GO term or protein classes. The enrichment for pArg and pSer/Thr is highlighted in turquois and pink respectively.

## Slide 22
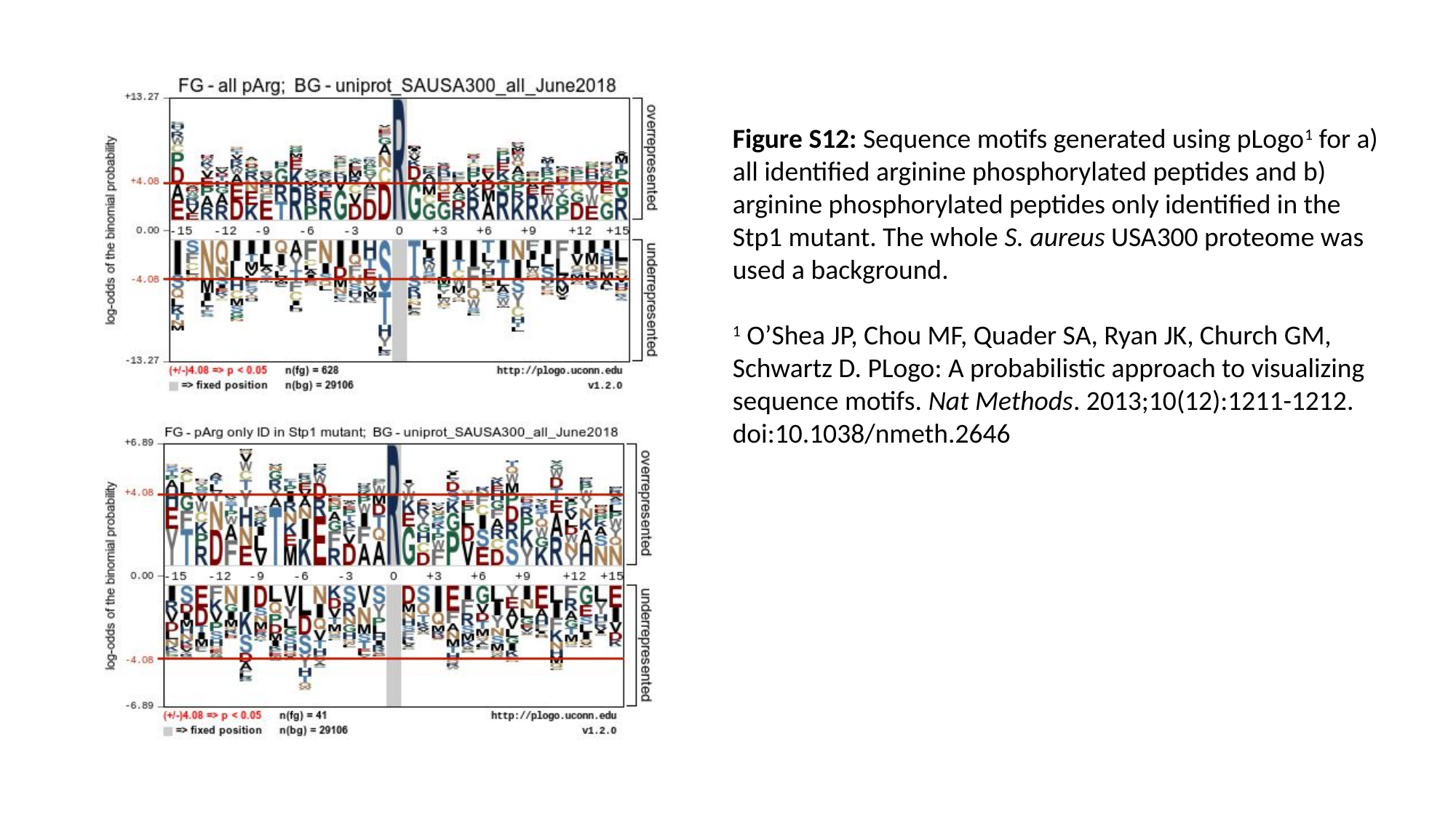

Figure S12: Sequence motifs generated using pLogo1 for a) all identified arginine phosphorylated peptides and b) arginine phosphorylated peptides only identified in the Stp1 mutant. The whole S. aureus USA300 proteome was used a background.
1 O’Shea JP, Chou MF, Quader SA, Ryan JK, Church GM, Schwartz D. PLogo: A probabilistic approach to visualizing sequence motifs. Nat Methods. 2013;10(12):1211-1212. doi:10.1038/nmeth.2646

## Slide 23
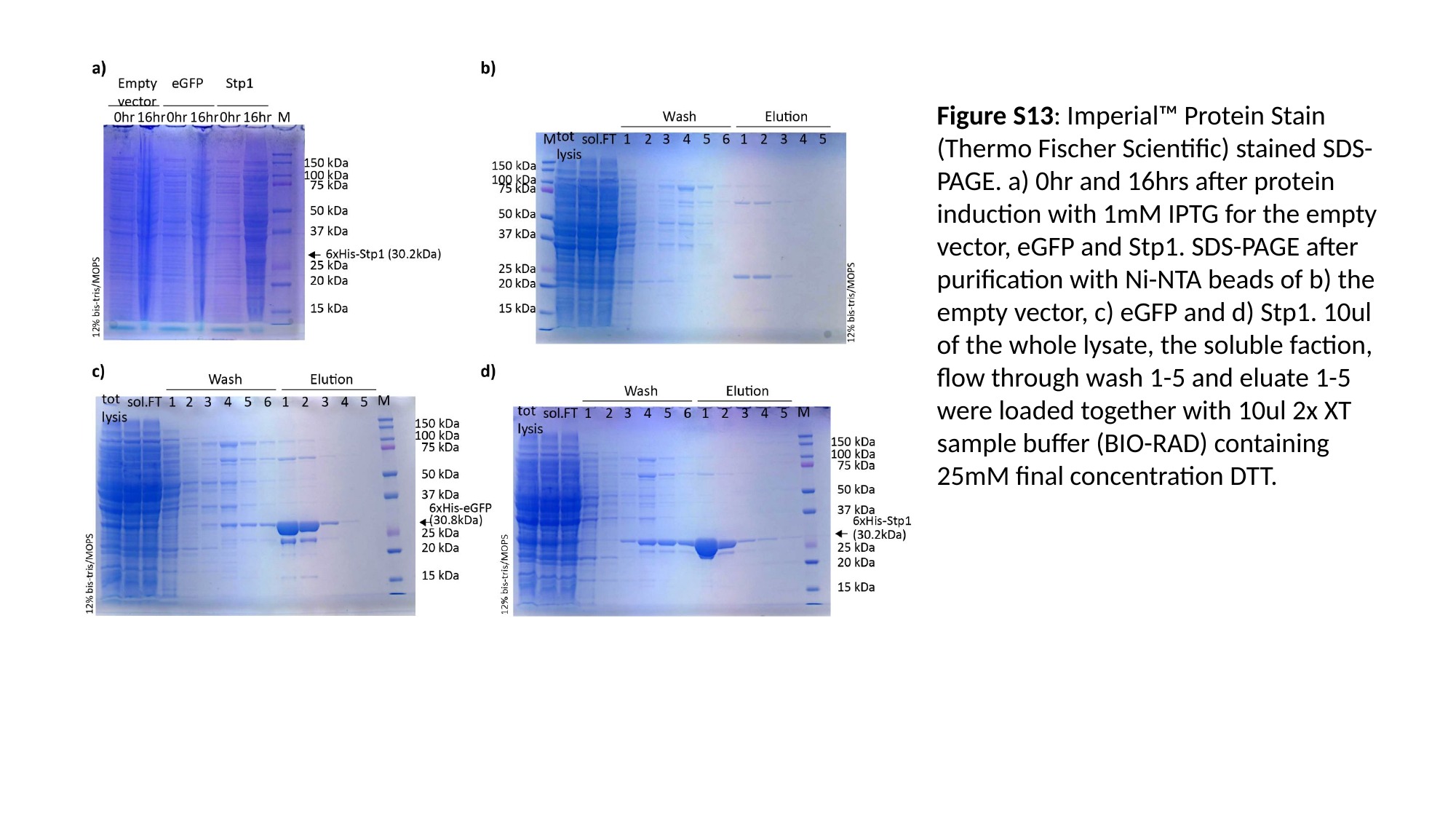

Figure S13: Imperial™ Protein Stain (Thermo Fischer Scientific) stained SDS-PAGE. a) 0hr and 16hrs after protein induction with 1mM IPTG for the empty vector, eGFP and Stp1. SDS-PAGE after purification with Ni-NTA beads of b) the empty vector, c) eGFP and d) Stp1. 10ul of the whole lysate, the soluble faction, flow through wash 1-5 and eluate 1-5 were loaded together with 10ul 2x XT sample buffer (BIO-RAD) containing 25mM final concentration DTT.
