# Supplementary material for: Widespread Arginine Phosphorylation in Staphylococcus aureus
Source: Mol Cell Proteomics. 2022 Apr 12;21(5):100232. doi: 10.1016/j.mcpro.2022.100232 (PMC9112008; doi:10.1016/j.mcpro.2022.100232)
Supplement: Supplemental Table S1 [file mmc2.docx]

**Supplemental Table 1:**

Fe3+-IMAC column enrichment gradient. Buffer A corresponds to the loading buffer (30 %ACN, 0.07 % TFA) and B to the elution buffer (0.3 % ammonia).

| **Time (min)** | **Flow rate (ml/min)** | **%A** | **%B** |
| --- | --- | --- | --- |
| **0 – 7.00** | 0.1 | 100 | 0 |
| **7.01 – 12.00** | 1 | 100 | 0 |
| **12.01 – 13.50** | 1 | 40 | 60 |
| **13.51 – 16.00** | 0.5 | 40 | 60 |
| **16.01 – 25.00** | 1 | 100 | 0 |
