# Supplementary material for: Widespread Arginine Phosphorylation in Staphylococcus aureus
Source: Mol Cell Proteomics. 2022 Apr 12;21(5):100232. doi: 10.1016/j.mcpro.2022.100232 (PMC9112008; doi:10.1016/j.mcpro.2022.100232)
Supplement: Supplemental Table S3 [file mmc4.docx]

**Supplemental table 3**

Chromosomal DNA templates from S. aureus and used primers

| # | sequence | name |
| --- | --- | --- |
| 2410 | acagcgCATATGataggtaaaataataaatgaacgatataaaattgtag | Fw_NdeI-PknB/Stk1 (SAUSA300_RS06025) |
| 2411 | acagcgGGATCCttatacatcatcatagctgacttctttttc | Rv_BamHI-PknB/Stk1 (SAUSA300_RS06025) |
| 2412 | acagcgCATATGctagaggcacaattttttactgata | Fw_NdeI-Stp1 (SAUSA300_RS06020) |
| 2413 | acagcgGGATCCtcatactttatcaccttcaatagccg | Rv_BamHI-Stp1 (SAUSA300_RS06020) |
